# Steroidal antibiotics are antimetabolites of Acanthamoeba steroidogenesis with phylogenetic implications[S]

**DOI:** 10.1194/jlr.M091587

**Published:** 2019-02-01

**Authors:** Wenxu Zhou, Emilio Ramos, Xunlu Zhu, Paxtyn M. Fisher, Medhanie E. Kidane, Boden H. Vanderloop, Crista D. Thomas, Juqiang Yan, Ujjal Singha, Minu Chaudhuri, Michael T. Nagel, W. David Nes

**Affiliations:** Department of Chemistry and Biochemistry,* Texas Tech University, Lubbock, TX 79409; Department of Microbiology and Immunology† Meharry Medical College, Nashville, TN 37208

**Keywords:** anti-amoeba drugs, suicide substrate, sterol biosynthesis

## Abstract

Pathogenic organisms may be sensitive to inhibitors of sterol biosynthesis, which carry antimetabolite properties, through manipulation of the key enzyme, sterol methyltransferase (SMT). Here, we isolated natural suicide substrates of the ergosterol biosynthesis pathway, cholesta-5,7,22,24-tetraenol (CHT) and ergosta-5,7,22,24(28)-tetraenol (ERGT), and demonstrated their interference in *Acanthamoeba castellanii* steroidogenesis: CHT and ERGT inhibit trophozoite growth (EC_50_ of 51 nM) without affecting cultured human cell growth. Washout experiments confirmed that the target for vulnerability was SMT. Chemical, kinetic, and protein-binding studies of inhibitors assayed with 24-*Ac*SMT [catalyzing C_28_-sterol via Δ^24(28)^-olefin production] and 28-*Ac*SMT [catalyzing C_29_-sterol via Δ^25(27)^-olefin production] revealed interrupted partitioning and irreversible complex formation from the conjugated double bond system in the side chain of either analog, particularly with 28-*Ac*SMT. Replacement of active site Tyr62 with Phe or Leu residues involved in cation-π interactions that model product specificity prevented protein inactivation. The alkylating properties and high selective index of 10^3^ for CHT and ERGT against 28-*Ac*SMT are indicative of a new class of steroidal antibiotic that, as an antimetabolite, can limit sterol expansion across phylogeny and provide a novel scaffold in the design of amoebicidal drugs. Animal studies of these suicide substrates can further explore the potential of their antibiotic properties.

As a result of evolution, sterol synthesis proceeds across eukaryotic kingdoms as a series of organized chemical steps affording flat-amphipathic Δ^5^-sterols that typically function as an architectural component of membranes (1–3). However, while there are relatively few sterolic genes involved in the conversion of 2,3-oxidosqualene to Δ^5^-sterol (4–6), there are several lineage-specific enzymes of distinct substrate preference and product specificity that, through their catalytic efficiency, establish the particular metabolite order preserved intact throughout nature (7–10). In some cases, organisms use linear pathways between metabolites that converge to produce the same final product, as in C_28_-ergosterol (11), whereas others diverge to yield chemical diversity, as in the epimeric pair of C_29_ α/β-sterols, stigmasterol and poriferasterol (**Fig. 1**; supplemental Fig. S1A, B) (2). Notably, the pattern of sterol complexity is determined by sterol methyltransferase (SMT), namely, 24-SMT (SMT1), which catalyzes the first C_1_-transfer reaction, and C28-SMT (SMT2), which catalyzes the second C_1_-transfer reaction. These enzymes distinguished mechanistically through a coupled methylation-deprotonation reaction that spawns Δ^24(28)^-olefins or Δ^25(27)^-olefins (supplemental Fig. S1C) capable of generating branched routes to products different in stereochemistry and supernumerary carbon (2, 12). While the sterol methylation reaction to form the C24-methyl or C28-methyl group in ergostane, stigmastane, and poriferastane skeletons requires common cationic intermediates, the possibility for natural substrate analogs potentiating disruptive SMT activity through misplaced intermediate reactivity remains enigmatic.

**Fig. 1.**
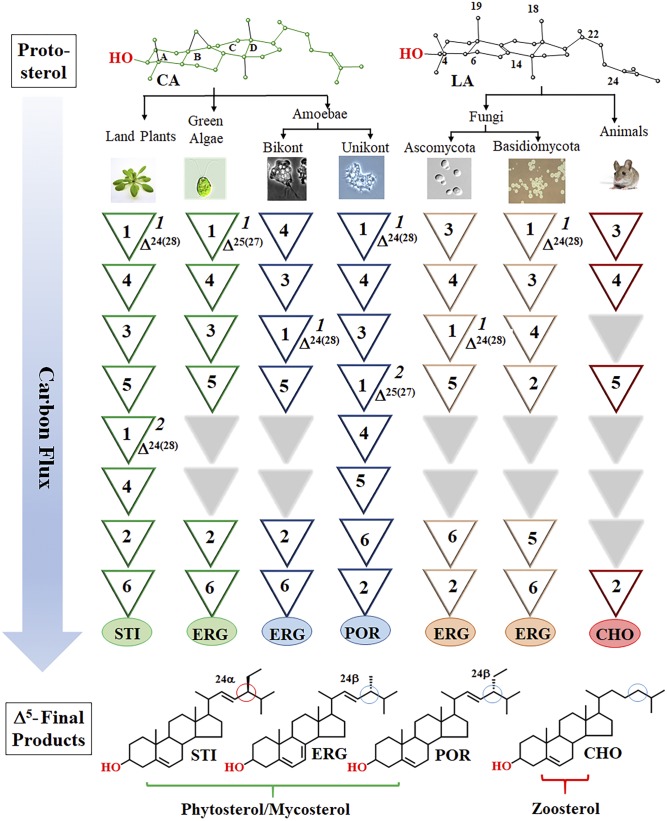
Variations in metabolite order in sterol biosynthesis. The numbers in inverted triangles indicate one or more enzymatic steps: step 1, sterol 24- or 28-methylation (*1*, 24-SMT; *2*, 28-SMT); step 2, sterol 25(27)- or 24(25)-, or 24(28)-reduction; step 3, sterol C14-demethylation; step 4, - sterol C4-demethylation; step 5, several biosynthetic steps that cluster after C14-demethylation involve B/C-ring metabolism of sterol C8 to C7 isomerization, sterol C5-desaturation, or C7-reduction to give the typical sequence of Δ^8^ to Δ^7^ to Δ^5,7^ to Δ^5^; step 6, sterol C22-desaturation (compare supplemental Fig. S1A). The Δ^5^-sterol products: STI, stigmasterol; ERG, ergosterol; CHO, cholesterol. The six representative sterol biosynthesis pathways are based on chemical identifications, labeling studies, and kinetic properties of pure enzymes isolated from land plants, green algae, bikont and unikont amoebae, ascomycete and basidiomycete fungi, and vertebrate animals (2, 8–11).

This revised understanding for plasticity in pathway topology raises new questions about the predicted sensitivities of pathogenic organisms to inhibitors of sterol biosynthesis and the rationale for divergence and disappearance of ancestral SMT in various lineages. In practice, one could identify antimetabolites, otherwise used as anticancer drugs (13), which target chokepoint enzymes in sterol biosynthesis through knowledge of endogenous metabolite structures that control growth. To date, all naturally occurring sterol intermediates are considered safe potential substrates for sterolic enzymes, so long as they possess the appropriate functional group(s) for turnover. Despite that, substrate analogs prepared with a toxic functional group to inactivate enzymes catalyzing the sterol C14-demethylation (CYP51) and sterol C24-methylation (SMT) reactions have proven harmful to protozoa viability by their ability to covalently bind CYP51 or SMT (14–17). These analogs may be considered a synthetic chemotype of antimetabolite for blocking ergosterol biosynthesis via protein alkylation.

During the course of our investigations of the *Acanthamoeba castellanii* responsible for blinding keratitis and granulomatous amebic encephalitis (16, 17), we observed natural C_27_- and C_28_-yeast sterols (supplemental Fig. S2) (10, 18) harboring the uncommon side chain diene group of Δ^22,24^, which can interfere with trophozoite growth by depleting cells of essential C_28_- and C_29_-phytosterols. In contrast, cholesterol supplementation to the medium does just the opposite; it can stimulate amoeba growth without effect on steroidogenesis. Intriguingly, feeding the steroidal Δ^22,24^-dienes to human epithelial kidney (HEK) cells has no effect on growth or cholesterol biosynthesis. The importance of these heretofore unrecognized observations is twofold. Foremost, Δ^22,24^-sterols, recognized now as a new class of antibiotic, could affect metabolic challenges as variables in sterol genealogy/biosynthesis driven by SMT gene gain. Likewise, these antimetabolites could replace intermediates or serve as product to compromise an evolving cholesterol biosynthesis pathway in animals capable of Δ^22^-introduction but constrained by reduced SMT gene expression or loss (19–21). Here, we report a comprehensive picture for SMT as a key mechanistic node to parasite termination and establish that substrate mimics synthesized in yeast as steroidal antimetabolites in Acanthamoeba potentially exist in the biosynthetic toolkit of other species to interfere with the normal metabolic processes within pathogenic organisms. Quite unexpectedly, we found the newly identified fungal antibiotics capable of protein alkylation in amoeba sterol biosynthesis provide a mechanism to limit the C_28_-/C_29_-sterol assemblage across phylogeny.

## MATERIALS AND METHODS

### Materials

The source of reagents and sterol substrates/standards cycloartenol (soybean seed), 24(28)-methylene lophenol (corn pollen), cyclolaudenol (*Chlamydomonas reinhardtii* cells), 24(28)-methylene cycloartanol (product of cloned soybean 24-SMT), cholesta-5,7,24-trienol (CTO) (*ERG*6 yeast mutant), cholesta-5,7,22,24-tetraenol (CHT) (*ERG*6 yeast mutant), ergosta-5,7,22,24(28)-tetraenol (ERGT) (product of cloned *Saccharomyces cerevisiae* SMT), cholesta-5,7,22-trienol (from incubation of GL7 yeast mutant with cholesta-5,7-dienol), ergosterol (*A. castellanii* cells), ergosta-5,7,24(28)-trienol (*Trypanosoma brucei* cells), 7-dehydroporiferasterol (*A. castellanii* cells), protothecasterol (*Prototheca wickerhamii* cells), and other ergostane, stigmastane and poriferastane monols from our sterol collection (19, 22, 23) shown in supplemental Table S1 (*A. castellanii *or *C. reinhardtii*). The [^3^H_3_-*methyl*]SAM (10 μCi/μM), [^2^H_3_-*methyl*]SAM (99.3% ^2^H_3_ enrichment-S/D/N isotopes; Ponte-Claire), and SAM chloride salt together with chromatographic materials, the Bradford protein assay kit, and the QuikChange site-directed mutagenesis kit (Strategene) were as described (19, 22). The [28-^2^H_2_]ERGT was prepared from deuterated SAM paired with CHT incubated against 24-*Ac*SMT.

### Metabolite analysis and structure identification

Amoeba cultures or HEK cells were harvested at one or more points during growth in the presence and absence of steroidal inhibitor. Cell pellets were split with an internal standard of 5α-cholestane added to one of the cell pellets for determination of sterol amounts in cells. Cells were saponified with 10% methanolic KOH and extracted with hexanes to yield a nonsaponifiable lipid fraction (NLF). The neutral lipids were analyzed by GC-MS and, for some samples, by TLC or HPLC-UV against cholesterol as a chromatographic reference. Ac strain ATCC 30010 was cultivated in tissue culture T-25 ml flasks, prepared with ATTC medium 712 in amounts of 10 ml, and then inoculated with pure trophozoites and incubated at 25°C statically with daily hand shaking. After a 4 day incubation, cells were pelleted and washed with phosphate buffer (10 ml ×3). HEK cells from ATCC were cultured in RPMI medium supplemented with 20% heat-inactivated fetal bovine serum in humidified atmosphere containing 5% CO_2_ as described (15). Growth was monitored by determining fresh weights of pellet and cell count with a hemocytometer. Pelleted cells were saponified in aqueous methanolic KOH (10% KOH, 5% water, 85% methanol, w/v) at reflux temperature for 1 h to produce a NLF of total sterol and a mixture of steroidal monols and steroidal diols.

Instrumental methods have been reported previously (11, 16, 17, 19). Briefly, proton and carbon NMR spectra were recorded in CDCl_3_ at ambient temperature using a 500 MHz spectrophotometer with the chemical shifts referenced to chloroform resonating at 7.265 ppm and reported as δ in parts per million. Mass spectra were obtained on a Hewlett-Packard 6890 GC-HP 5973 MSD instrument (electron impact, 70 eV; scan range, *m/z* 50–550 amu). HPLC was carried out at room temperature using a Phenomenex Luna C18-column (250 mm × 4.6. mm × 5 μM) connected to a diode array multiple wavelength diode array detector with 5% aqueous methanol as eluant. Capillary GC (0.25 mm internal diameter, by 30 m fused silica column coated with Zebron ZB-5 from Phenomenex) was operated at a flow rate of He set at 1.2 ml/min, injector port set at 250°C, and a temperature program of initial 170°C, held for 1 min and increased at 20°C/min to 280°C. Retention times of sterols were normalized to their retention time relative to that of cholesterol in GC (RRTc) of 13.8 min (or a bit longer to 14.5 min subject to column clipping) or in HPLC (α_c_) of 20 min. and were compared with those of authentic standards in our sterol collection.

In sterol analysis, product distributions were determined by approximate integration of chromatographic peaks. The sterol was routinely examined as the 3-OH compound; but in some cases to show the number of hydroxyl groups in the structure, total sterol in the NLF was prepared as the TMS derivative as follows: The extracted sterol was converted to the TMS ester using 15 μl of *N*,*O*-Bis(trimethylsilyl)trifluoroacetamide with trimethylchlorosilane (99:1) and 15 μl of pyridine as catalyst at room temperature for 5 min. The resulting TMS derivative (1 μl) was injected directly into the gas chromatograph mass spectrometer for analysis.

### Time-kill and washout assays

Minimum amoebicidal concentration (MAC) (defined as the lowest concentration of inhibitor with no visible live trophozoites), IC_50_ (50% effective dose value of inhibitor), and time-kill assay were determined at 25°C in 24-well plates containing a total volume of 1 ml as previously described (17). Experiments were performed in triplicate (or in duplicate with excellent agreement between biological replicates of less than 10% difference in cell count) using a light microscope to count cells and trypan blue staining to establish cell death. Target vulnerability can be assessed through cell washout experiments where free drug (or in our case, steroidal inhibitor) is “removed” from the system by washing or dilution, while continued occupancy of the drug after washing can be due to protein alkylation. Here, 25 ml T-flasks supplemented with 10 ml of medium and treated with 10 μM of exogenous sterol were inoculated with 5 × 10^4^ cells/ml and cultured for 96 h to early growth arrest, which for control yields 1–5 × 10^6^ cells/ml. Arrested cultures were pelleted. A 10-fold serially diluted suspension was used as inoculum for further growth experiments in 24-well plates prepared as in the MAC experiments. An untreated sample was used as a negative control. At 96 h growth, following inoculation of 10^6^ cells/ml, 10^5^ cells/ml, 10^4^ cells/ml, and 10^3^ cells/ml, cell number was determined using a hemocytometer. Experiments were performed in triplicate (N = 3 ± 10%). In a separate experiment, [28-^3^H_2_]ERGT, prepared from CHT and [^3^H_3_-*methyl*]SAM against cloned 24-*Ac*SMT then purified by HPLC, was added to cell cultures at 20 μM. The cultures were inoculated with 5 × 10^4^ to 1 × 10^5^ cells/ml and grown for 4 days, and then the cells were pelleted for further processing.

### Kinetic and product determination of *Ac*SMT catalysis

General methods for heterologous expression, lysate preparation, steady-state experiments for substrate conversion to product, and inhibitor (IC_50_) determination were as reported (16, 17). Assays were performed at 35°C for 45 min (predetermined initial velocity conditions) in triplicate with less than 10% deviation. Conversion of the IC_50_ value (based on dose-response plots of increasing inhibitor concentration ranging from 4 to 150 μM against saturating amounts of 100 μM native substrate cycloartenol for 24-*Ac*SMT; *K_m_* = 48 μM or 24(28)-methylene lophenol for 28-*Ac*SMT; *K_m_* = 25 μM) and 150 μM SAM to *K_i_* value was accomplished using the Cheng-Prussoff equation (12). To promote maximum conversion of substrate by SMT catalysis, preparative incubations were carried out overnight with saturating sterol (100 μM) and excess SAM (300 μM) against 2.5 or 5.0 mg/ml total lysate protein.

### Site-directed mutagenesis and *Ac*SMT purification

The *A. castellanii* sterol C24-methyltransferase genes (24-SMT, XP_004336540 and 28-SMT, XP_004335307) were synthesized by Eurofin MWG Operon (Huntsville, AL) incorporating an Nde1 restriction site at the 5′ end and a BAMHI restriction site at the 3′ end of the open reading frames. Genes were cloned in pET11a expression vector (Novagen, Madison, WI). Gene integrity was verified by PCR using gene-specific primers and by DNA sequencing. To ease protein purification, two new constructs of 24-*Ac*SMT and 28-*Ac*SMT were prepared using pET30b vector with 6xHis_3_ tags on both termini and an S-tag on the N terminus to enhance protein solubility. The vectors were transferred to *E. coli* BL21 (DE3) cells for protein expression. Cells were grown in Luria-Bertani broth (pH 7.5) supplemented with kanamycin (50 μg/ml) and grown for 3.5 h at 30°C at 200 rpm shaking. Expression was induced by the addition of IPTG (400 uM) followed by incubation for another 18 h. Protein isolation and lysate preparation were performed according to our previous methods (16–18). The activities of the new recombinant SMTs were similar to those of their nontagged counterparts, indicating that the native conformation of wild-type enzyme was retained following protein expression of His_12_-tagged SMT.

SMT was purified using Ni-NTA chromatography as follows: The total broken cell soluble preparation (25,000 g supernatant) of 5–20 mg total protein was loaded onto HisPur NTA resin (2 ml) packed into a HisPur Ni-NTA spin column (Thermo Scientific) and washed with high salt buffer to remove unspecific binding, then low salt washing to remove non-His-tagged protein (total 200 ml). The SMT was eluted with a step-wise gradient by elution buffer containing imidazole concentrations of 50, 75, 100, and 150 mM (2 ml each), respectively. The levels of expression, purity, and size of the recombinant protein were monitored chromatographically on a 10% SDS-PAGE gel electrophoresis followed by Coomassie Blue R250 staining. Total protein concentrations were determined by the Bradford method with commercial reagents (Bio-Rad) using bovine γ-globulin as standard. Preliminary evaluation of the enzyme activity from lysate preparation by GC-MS analysis confirmed that the cloned enzymes harboring the two His-tags were equally active to those of wild-type protein. The molecular mass of the new proteins differed: wild-type 24-AcSMT, 39.0 kDa and 28-AcSMT, 39.3 kDa versus His_6_-constructs of 24-AcSMT, 45.7 kDa and 28-AcSMT, 46.2 kDa. Point mutations were generated using the QuickChange II site-directed mutagenesis kit (Agilent Technologies) according to the manufacturer’s instructions from 24-*Ac*SMT and 28-*Ac*SMT wild-type plasmids that contained two His_6_-tags, one at the N terminus and the other at the C terminus. The oligonucleotide sequences of mutagenic primers are listed in supplemental Table S2. Mutations were introduced at position 60 of 24-AcSMT and position 64 of 28-*Ac*SMT of Phe or Leu to replace the conserved amino acid tyrosine (supplemental Fig. S10, supplemental Table S2). In contrast to wild-type SMTs, which expressed to similar levels, the His_12_-tagged 28-*Ac*SMT expressed to about 50% level of 24-*Ac*SMT (supplemental Fig. S11).

### Inactivation studies

Partitioning experiments to measure the C24-methyl monol sterol [ergosta-5,7,22,24(28)-tetraen-3-ol and ergosta-5,7,22,25(27)-tetraen-3-ol] to C24-methyl diol sterol [ergosta-5,7,23(24),3,22-diol] ratio were carried out in standard assay conditions at 10 min, 20 min, 45 min, 1 h, 2 h, 3 h, 6 h, and 24 h using 100 μM substrate and 150 μM SAM. The reaction was terminated by addition of methanolic KOH and the total sterol recovered in the usual manner for GC-MS analysis. Quantification of the monol and diol products was determined by measuring the abundance of each ion (expressed as area under the curve) for the relevant molecular ion in the mass spectrum generated by the selected ion monitoring (SIM) technique at the high mass end for M^+^ 380 (substrate), 394 (product), and 412 (product) amu, respectively. The enzyme-generated sterol composition of the incubation mixture was analyzed by GS-MS.

To measure time- and concentration-dependent generation of C24-methyl monol C_28_-sterol [ergosta-5,7,22,24(28)-tetraen-3-ol and ergosta-5,7,22,25(27)-tetraen-3-ol] to C24-methyl diol sterol [ergosta-5,7,23(24),3,22-diol] from CHT incubation with 28-*Ac*SMT, assays were carried out under initial velocity conditions using the standard protocol at 10 min, 20 min, 45 min, 1 h, 2 h, 3 h, 6 h, and 24 h of lysate: SMT (approximately 100 μg SMT per assay) was varied from 5 to 50 μM against fixed CHT at 100 μM and SAM at 150 μM. These conditions limit SAM and therefore limit the enzyme from generating C_29_-sterol, thereby simplifying the analysis. Time-course studies were carried out from 0 to 300 min. The reaction was terminated by addition of methanolic KOH and the total sterol recovered in the usual manner for GC-MS analysis. Quantification of the monol and diol products was determined by measuring the abundance of each ion (expressed as area under the curve) for the relevant molecular ion in the mass spectrum generated by the SIM technique at the high mass end for M^+^ 380 (substrate), 394 (product), and 412 (product) amu, respectively.

For separate tests of enzyme inactivation, small scale (600 ml) incubations of SMT performed under standard assay conditions against 100 μM CTO as preferred substrate because it can bind effectively to both 24-*Ac*SMT and 28-AcSMT and is close in structure to the analog inhibitor. Substrate protection against SMT2 was determined by preincubation of CTO or 24(28)-methylene lophenol at 50 μM and 100 μM concentrations for 10 min, and then 20 μM of analog CHT was added to the reaction vial and incubated for another 1 h at 35°C. The reaction was terminated by the addition of methanolic KOH. The resulting organic extract was analyzed for product formation by GC-MS.

### Covalent binding experiments

A soluble preparation containing 100 μM ERGT or DHL, 300 μM SAM amended with catalytic amounts of [^3^H_3_-*methyl*]SAM (3 × 10^6^ dpm), and 5 mg total protein (approximately 250 μg 28-SMT or 500 μg 24-SMT) was incubated overnight under standard assay conditions, affording a ^3^H-analog-SMT complex. The sterol-protein complex was purified using Ni-NTA chromatography eluted with imidazole in 5 ml fractions as described above. An aliquot of each fraction (200 μl) was added to scintillation fluid and counted on a scintillation counter. Most radioactivity (>80–95%, depending on the source of SMT) was recovered in the wash.

### Proteomic analysis

His_12_-tagged recombinant wild-type 28-*Ac*SMT and 28-*Ac*SMT complexed with analog were purified using the standard protocol. The imidazole was removed from the eluted protein solution and the buffer was changed to 20 mM Tris-HCl (pH 8.0) using ultrafiltration. After buffer change, the protein was diluted to 1 mg/ml (based on the calculation of absorption at optical density at UV 280) and incubated with ERGT and SAM using the standardized assay condition as described (22). A parallel assay with sterol and no SAM was used as control. After overnight incubation at 35°C, the assay mixture was subjected to Ni-NTA to remove loosely bound sterol and SAM. The pure alkylated and control proteins were concentrated followed by a buffer change to 50 mM ammonium bicarbonate buffer (pH 8.0). The protein concentrations were determined at OD_280_ and purity confirmed by SDS-PAGE. The molecular mass of both native and alkylated proteins was determined using an MDS SCIEX 4800 MALDI TOF/TOF™ analyzer (Applied Biosystems, Foster City, CA) with sinapinic acid as matrix for assisting ionization.

## RESULTS

### Unmasking amoeba steroidogenesis vulnerability to substrate mimicry

**Fig. 2.**
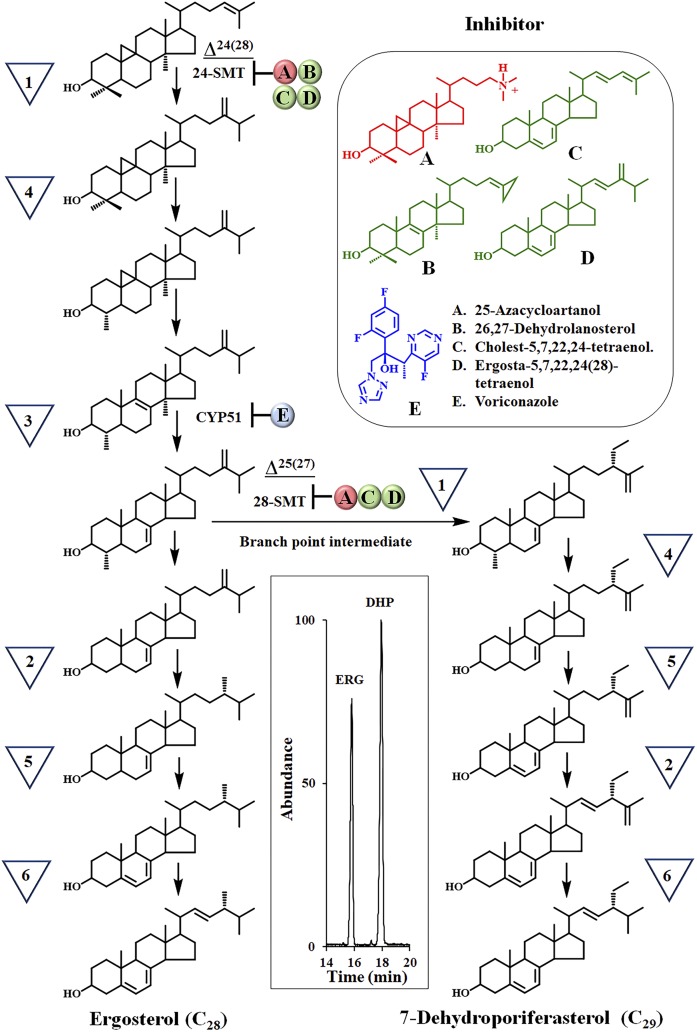
*A. castellanii* sterol biosynthesis pathway. The inverted triangles represent biosynthetic steps as defined in Fig. 1. Inhibitors were tested against Ac cell growth and isolated enzymes, SMT and CYP51, as reported in (16, 17) and results of studies herein. The inset is a GC profile of total sterols isolated from 4 day growth-arrested trophozoites. ERG, ergosterol; DHP, 7-dehydroporiferasterol.

Our previous studies of *A. castellanii* cells revealed that: *i*) two functional classes of SMT can operate in tandem, such that the Δ^24(28)^-olefin pathway catalyzed by SMT1 precedes the Δ^25(27)^-olefin pathway catalyzed by SMT2; *ii*) C_29_-sterol biosynthesis proceeds as a branch pathway of the canonical C_28_-sterol biosynthesis pathway to ergosterol (**Fig. 2**); and *iii*) the C_28_- and C_29_-sterol composition can undergo marked changes as trophozoites (that synthesize ergosterol and 7-dehydroporiferasterol) differentiate into resting cysts (that synthesize brassicasterol and poriferasterol) or lyse/die (from synthesis of 6-methyl aromatic sterols) (16, 17). We further discovered that the proportion of C_28_- and C_29_-sterols at growth arrest can change by supplementing the medium with either a tight binding inhibitor, such as 25-azacycloartanol, or a suicide substrate inhibitor, such as 26,27-dehydrolanosterol, that selectively target the *Ac*SMTs that, thereby, kill trophozoites (Fig. 2, supplemental Fig. S1C).

Initially, 10 μM of cholesterol in the form of fetal bovine serum or 10 μM solubilized in DMSO were tested against Ac trophozoites cultured in T-flasks and observed to stimulate growth (supplemental Fig. S3). GC-MS analysis of 4 day growth-arrested Ac cultures showed that cholesterol from the serum-treated cells represents about 15% total sterol, while the endogenous amoeba sterol composition remain unchanged. CTO supplemented to the medium at 10 μM had no effect on growth or amoeba steroidogenesis. In contrast, Ac cultured on 10 μM yeast sterol, CHT or ERGT, for 4 days inhibited cell proliferation, affording approximately 15% (±5%) of the cell number or fresh weight of the pellet relative to control. GC-MS analysis of total sterols showed the C_28-_ergosterol to C_29_-7-dehydroporiferasterol ratio in the control was 44:55 [Fig. 2, inset (GC profile)], while the C_28_-ergosterol to C_29_-7-dehydroporiferasterol ratio in CHT- and ERGT-treated cells was slightly modified, 36:64 and 38:62, respectively; the amount of endogenous ergosterol and 7-dehydroporiferasterol in treated cells decreased by approximately 15%, while approximately 10% of the dietary sterol was accumulated into the cell. Although these growth experiments of treated cells were repeated several times and the sterol composition of 4 day cells analyzed by GC-MS, in no case was there detectable substrate accumulation of either cycloartenol or 24(28)-methylene lophenol or of steroidal diol formation, even when using SIM of the relevant ions to check for trace sterol in the chromatogram.

The [28-^3^H_2_]ERGT was prepared enzymatically from CHT and ^3^H-SAM (specific activity 1 × 10^5^ dpm/70 μg) and incubated with trophozoites cultured as above for 4 days. The radioactivity of the resulting cell pellet was initially 1 × 10^4^ dpm. After five washes against phosphate buffer, the cell pellet radioactivity dropped to 0 dpm, showing excess ^3^H completely removed by the washout. The pellet was then extracted with methanol/chloroform to remove ^3^H-sterol accumulated by cells yielding 2.6 × 10^3^ dpm. These results indicate approximately 9% uptake of ^3^H-sterol into the cell, which is in good agreement with the quantification of ERGT into the cells by GC-MS analysis above. The pellet was subsequently saponified in aqueous methanol KOH and then extracted with hexane yielding an organic extract of 286 dpm, consistent with protein alkylation. In a separate experiment, we investigated to determine whether an extended duration of action can be observed through the growth response even when the drug has been cleared. Here, control and treated cells were supplemented with 10 μM ERGT and incubated for 4 days as in the radioactivity experiment. The resulting cell pellet was washed to remove unbound analog, and then divided into three samples of inoculum of 1 × 10^3^ cells/ml, 1 × 10^4^ cells/ml, and 1 × 10^5^ cells/ml added to 1 ml of medium in a 24-well plate system. The cells were cultured for 96 h to permit adequate time for 100% growth recovery to 1 × 10^6^ cells/ml. At the conclusion of the growth experiment, the percentage of cells in the treated cultures compared with control was 0, 20, and 40%, respectively. Thus, selectivity for target engagement was confirmed.

**Fig. 3.**
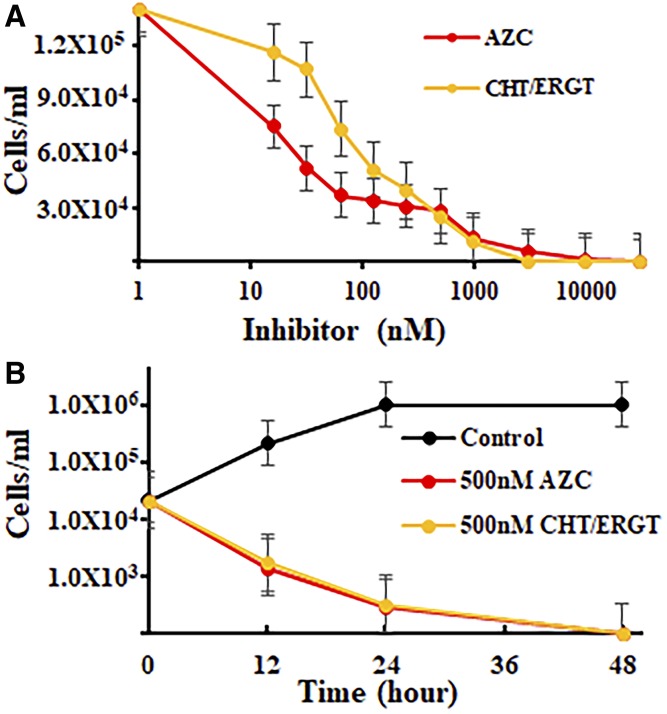
*A. castellanii* growth response to the suicide substrates, CHT and ERGT, in comparison to our published report of inhibition by 25-azacycloartanol (AZC) (17). The SEMs are less than 5% of three independent experiments. Ac trophozoites (90–95% pure) were inoculated into 24-well plates as described in the Materials and Methods at varied concentrations of inhibitor (A) or at fixed concentration of inhibitor (B).

Given these results, we determined the Ac growth response to CHT and ERGT using the standard protocol of amoebae seeded into 24/96-well plate systems. We used this system successfully in our evaluation of medical azoles and steroidal transition state analogs that bind CYP51 and SMT (16, 17, 24). Both CHT and ERGT generated IC_50_s of approximately 51 nM and a MAC of approximately 5 μM, while 25-azacycloartanol, used as reference (17), inhibits growth with similar potency (**Fig. 3A**). Alternatively, time-kill studies of CHT or ERGT led to cell death by 48 h (Fig. 3).

### CHT and ERGT: neutral effect on cultured HEK cell growth and cholesterol biosynthesis

CHT and ERGT were incubated in HEK cells and showed no effect on cell proliferation to 40 μM. GC-MS analysis of 40 μM treated HEK cells showed that both dietary supplements were accumulated by cells (supplemental Fig. S3). However, while neither compound had an effect on cholesterol biosynthesis, both of them were metabolized to a steroidal triene, as noted by a gain in two hydrogen atoms (M^+^ 380 to M^+^ 382) (supplemental Fig. S5) (17–25). MS showing loss of the *m/z* 143/157 fragment for the nucleus Δ^5,7^-system (2, 25) and ions at 109/269/271 amu showing retention of the conjugated diene side chain system support that metabolism occurred in the nucleus. Moreover, the chromatographic mobility of cholesta-5,22,24-trienol is different from authentic samples of 5,7,22-trienol and CTO, while the chromatographic mobility of ergosta-5,22,24(28)-trienol is different from authentic samples of ergosta-5,7,22-trienol and ergosta-5,7,24(28)-trienol (supplemental Table S1). These results suggest that the cholesterol biosynthesis pathway in HEK cells utilizes a Δ^7^-sterol reductase enzyme in metabolism of CHT and ERGT, while the Δ^24^-reductase fails to bind the two yeast sterols productively. Comparing the IC_50_ growth values for CHT or ERGT supplied to Ac with the growth response of HEK cells to CHT or ERGT at 40 μM, the highest concentration tested affording no effect on growth, yields a selective index of approximately 10^3^, which shows that the steroidal inhibitors are of equal potency to azolic CYP51 inhibitors as anti-amoeba agents (24).

### Leveraging SMT catalysis for steroidal antibiotic characterization

With the availability of our previously cloned 24-*Ac*SMT and 28-*Ac*SMT (9), we could determine the structural specificity of the enzymes to substrate analogs and the sterol methylation reaction pathway that determine turnover versus protein alkylation. As a control, we started with 24-*Ac*SMT exposed to CTO, which does not possess a Δ^22^-bond. GC-MS analysis of the sterol region from 13 to 20 min revealed that the substrate converts to a single product, ergosta-5,7,24(28)-trienol **9** (100%) (supplemental Fig. S6). However, 28-*Ac*SMT incubated with CTO generates C28- and C29-sterol products representing the first and second C_1_-transfer reactions, consistent with the established SMT2 substrate acceptance of CTO (9). The product of the first C_1_-transfer reaction is the same as in 24-*Ac*SMT catalysis. The C28-product then undergoes a second C28-methylation yielding a triplet of C_29_-steroidal monol **10**, **11**, and **12** products with the Δ^25(27)^-olefin **10** representing the major product (supplemental Figs. S6, S7). To determine whether the individual C_1_- and C_2_-transfer reactions are dependent on a common cationic intermediate, we conducted an isotopically sensitive branching experiment of 24-*Ac*SMT and 28-*Ac*SMT incubated with CTO paired with [^2^H_3_-*methyl*]SAM. Against 24-*Ac*SMT, CTO converts to [28-^2^H_2_]ergosta-5,7,24(28)-trienol in 100% yield. However, against 28-*Ac*SMT, the amount of enzyme-generated C_29_-sterol is reduced markedly in the product mixture to 50% and the [28-^2^H]C_29_-olefinic product ratio altered such that Δ^24(28)^-olefin formation **11**/**12** is suppressed, while Δ^25(27)^-olefin formation **10** is increased (supplemental Figs. S1C, S6). The results show a strong kinetic isotope effect on C28 deprotonation mated to the intermediate C24-cation that determines product turnover in the step-wise C_2_-transfer reaction. The inability to detect a KIE in the first C_1_-transfer reaction is consistent with a nonstop methylation-deprotonation reaction pathway at this level of sterol methylation. Intriguingly, the kinetic disruption did not alter branching in the direction of enzyme inactivation from which steroidal diol should be evident by GC-MS analysis of the saponified sample.

**Fig. 4.**
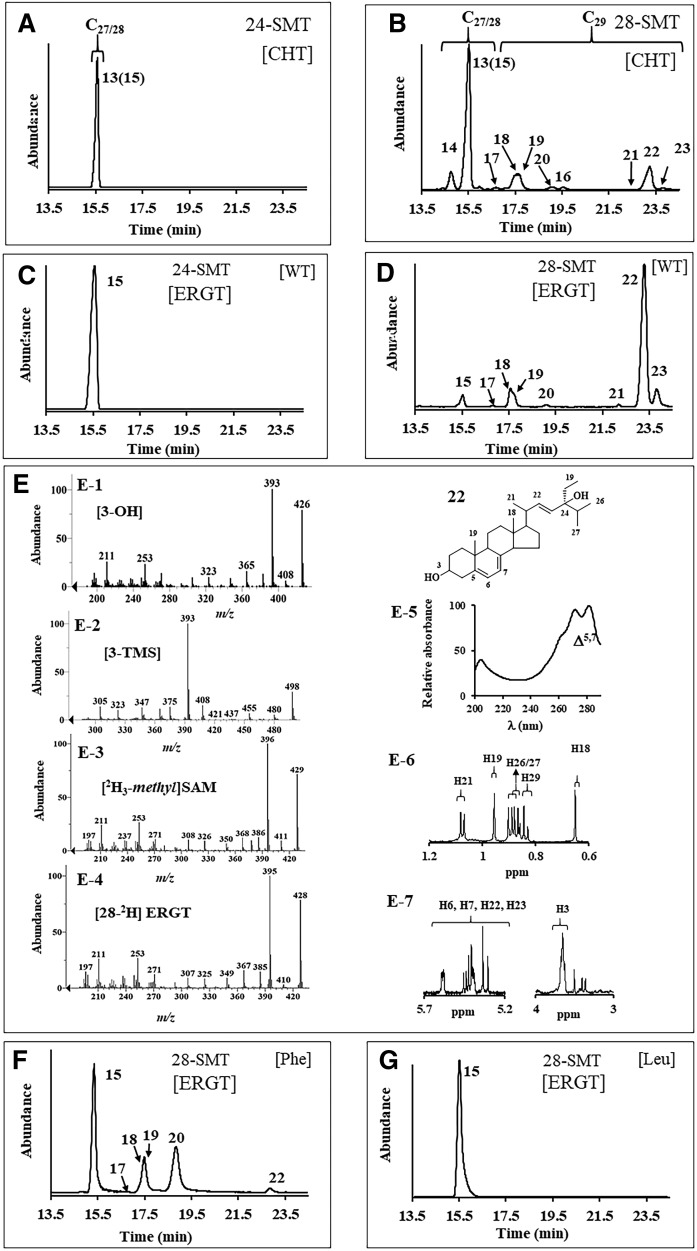
Chromatographic and spectral analysis of SMT-generated C_28_- and C_29_-sterol products. A–D: GC profile of total sterol from 24-*Ac*SMT or 28-*Ac*SMT incubated with CHT or ERGT. E: Mass spectrum of compound **22** prepared as indicated for panels E-1 to E-4. Panel E-5 is the UV spectrum of **22**, while panels E-6 and E-7 are the NMR with assignment of **22**. F, G: GC profiles for total sterol generated from mutant 28-AcSMT assayed with SAM and ERGT.

Next, 24-*Ac*SMT and 28-*Ac*SMT were assayed with CHT and ERGT. GC-MS analysis of enzyme-generated sterol profiles show that 24-*Ac*SMT converts CHT to ERGT in 50% yield (based on the ratio of molecular ions *m/z* 380 and 394, **Fig. 4A**) without any accompanying byproduct, in agreement with our incubation of CHT with yeast *S. cerevisiae* SMT, which catalyzes the Δ^24(28)^-olefin pathway (data not shown) yielding ERGT in 100% yield; ERGT is not productively bound to either 24-*Ac*SMT (Fig. 4C) or *Sc*SMT (data not shown). Alternatively, 28-*Ac*SMT converts CHT to a set of expected C_28_- and C_29_-sterols in the “sterol region” of the chromatogram between 13 and 20 min and a second set of unexpected peaks that elute late in the GC chromatogram between 19.5 and 24 min (Fig. 4B). Mass ions for these new compounds corresponded to hydroxylation derivatives suggesting C_28_- and C_29_-steroidal Δ^22^-diols (25) (supplemental Fig. S5). These diols appear only in GC analysis following saponification of cell extract, while chloroform-methanol extractions yield a mixture of steroidal monols. Consequently, we have adopted the SMT-generated diol product as an indicator of protein alkylation.

**Fig. 5.**
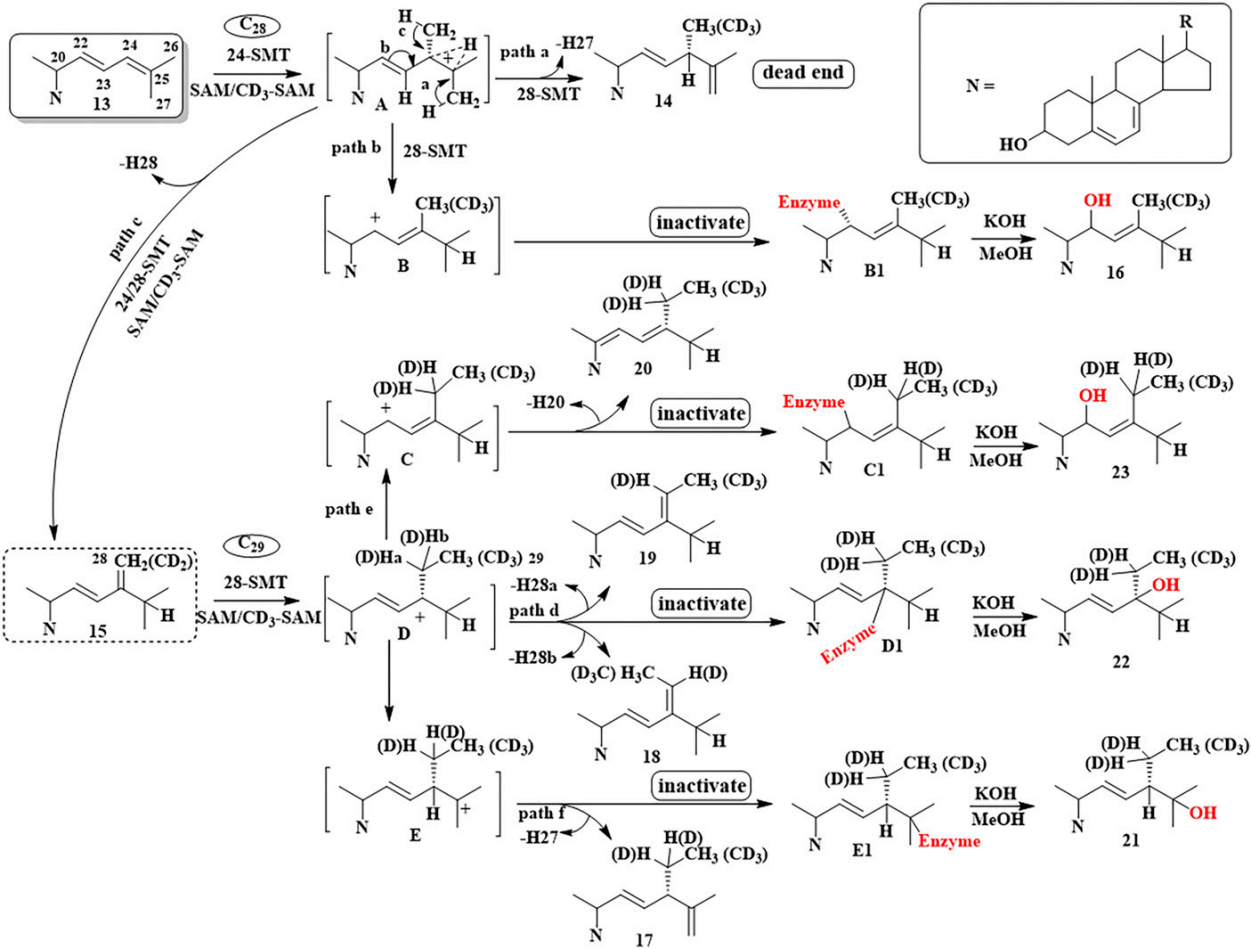
Proposed sterol methylation pathways of Δ^22,24^-sterol substrates incubated with 24-SMT and 28-SMT enzymes. As discussed in the text, SMTs that favor the Δ^24(28)^-olefin route are more likely to forward these substrates to productive products, while SMTs that favor the Δ^25(27)^-olefin route are more likely to forward these substrates into protein alkylation.

Incubation of 28-*Ac*SMT with CHT led to six steroidal monols eluting in GC between 14 and 19.5 min, each of which possess a similar parent M^+^ peak, *m/z* 394 (from C24-methylation) or 408 (from C28-methylation). In GC, the isomeric C_28_- and C_29_-sterol products showed relevant ions at M^+^, M^+^-CH_3_, M^+^-H_2_O, and M^+^-CH_3_-H_2_O, and a base peak diagnostic of their side chain structures as reported (2, 23, 25) (supplemental Fig. S5). MS and GC RRTc values of compounds **14** and **15** matched those of authentic standards (16, 17, 26, 27), while the identification of the other compounds required more analysis. In HPLC, **17**, **18**, **19**, and **20** elute as a wide-fraction α_c_ 1.65–1.70. The sterols possessed a similar fingerprint λmax at 282 nm typical of a Δ^5,7^-system in the nucleus. The UV spectrum for **17** also possessed end absorption showing the side chain double bonds were not in conjugation, while the UV spectra for **18**, **19**, and **20** possessed an additional λmax at approximately 230 nm, indicative of a conjugated double bond in the side chain, which, for biosynthetic reasons, placed the double bonds toward C20 (supplemental Table S1). Product **20** possessed an electron impact mass spectrum showing an ion at *m/z* 137 for fragment C_10_H_17_ (not present in MS of **17**, **18**, or **19**) representing cleavage of the side chain through the C17(20) bond due to a double bond at C20(22) (supplemental Fig. S5). This product **20** could be unambiguously distinguished from its isomers, **18** and **19**, through incubation of 28-*Ac*SMT with [28-^2^H_2_]ERGT and SAM. Enzyme-generated 28-^2^H_2_ **20** retains two deuterium atoms in the side chain (M^+^ 410), proving that deprotonation does not occur at C28, while 28-^2^H **18** and 28-^2^H **19** each have lost one deuterium atom in the side chain, proving deprotonation at C28 (M^+^ 409) (supplemental Fig. S8). For mechanistic reasons, sterol methylation to form **18** and **19** follows the Δ^24(28)^-olefin pathway while **20** formation involves a new route (**Fig. 5**).

In the case of the putative steroidal diols eluting late in the GC trace, MS analysis of **21**, **22**, and **23** following incubation of 28-*Ac*SMT with ERGT paired with [^2^H_3_*-methyl*]SAM demonstrated that these products originated catalytically in methylation by SAM (supplemental Figs. S8, S9). In the incubation of 28-*Ac*SMT with CHT, a peak appeared in the GC chromatogram for **16**, previously identified as a product of CHT incubation with *T. brucei* SMT that catalyzes the Δ^25(27)^-olefin pathway (23, 27). In the incubation of 28-*Ac*SMT with CHT or ERGT, identification of the late eluting peaks in GC corresponding to **21**, **22**, and **23** was a bit more challenging because no reference standards are available for these compounds. Their retention time and mass of *m/z* 426 are consistent with formation of a C_29_-steroidal diol of molecular formula C_29_H_46_O_2_ (25–27). In HPLC, **21** elutes at α_c_ 0.52, **22** elutes at α_c_ 0.54, and **23** elutes at α_c_ 0.50 before ERGT, which elutes late at α_c_ 1.35. In UV, **21**, **22**, and **23** possess similar spectra showing a typical finger print for a Δ^5,7^-system in the sterol nucleus of λmax of 282 nm (Fig. 4, panel E-5).

The identity of the isomers could be firmly established by a combination of approaches in which the products were TMS-derivatized, isotopically labeled at C28 with one or two deuterium atoms, or analyzed by NMR: Thus, the TMS-derivative of **21** and **22** possessed *m/z* M^+^ 498, while **23** possessed M^+^ 570 (Fig. 4), consistent with **21** and **22** having one secondary and one tertiary alcohol in the structure, while **23** has two secondary alcohols in the structure. MS of products of 28-*Ac*SMT incubated with SAM paired with [28-^2^H_2_]ERGT showed *m/z* M^+^ 428 for compounds **21**, **22**, and **23**, indicating that no C28-deprotonation occurred (Fig. 4; supplemental Figs. S8, S9). The ^1^H-NMR spectrum of **22** indirectly located the tertiary OH group at C24 rather than at C25 through relevant doublets for C26/C27 resonating at 0.87 (d) and 0.89 (d). Of the four olefinic protons, two in the side chain were coupled to one another (*j* =15.0 1H, dd; 15.4 1H, dd) indicating the presence of an *E*-disubstituted alkene originating in the substrate. A signal for the C3β-OH group (Fig. 4, panels E-5–E-7) and signals for side chain methyl/methylene groups at H18 (singlet), H19 (singlet), H21 (doublet), and H29 (triplet) (Fig. 4) confirm the identification of **22** distinct from **21**. Structure **23** was initially distinguished from **21** and **22** by its TMS-derivative. MS of **23** showed a fragment for side chain cleavage at *m/z* 127 consistent with the location of C22 in the side chain (25, 27). NMR analysis provided additional proof of structure showing relevant signals for H18 0.636 (s), H19 0.954 (s), H21/H26/27 0.96 to 1.019 (m), H22 (bearing OH) 5.320 (d), and H23 5.30 (m). On the basis of these results, several new inactivation metabolites have been characterized and a new sterol methylation pathway has been proposed that recognize alternate trajectories for the methylated Δ^22,24^-intermediate to proceed, either to turnover or covalent bind to enzyme (Fig. 5).

### Active-site mutagenesis reveals a residue switch that models reaction channeling

Recent site-directed mutagenesis experiments of SMT enzymes have demonstrated the importance of tyrosine residues in the substrate binding segment Region 1 lined with aromatic residues [Region 1, Y**X**YWGWG**XX**FHF (supplemental Fig. S10)], and together with homology modeling of the SMT active site suggested the tyrosine60/64 in the amoeba SMTs can play a role in binding and catalysis (27, 28). Specifically, this conserved residue may stabilize carbocation intermediates generated during sterol methylation through cation-π interactions that channel deprotonation routes into Δ^25(27)^- or Δ^24(28)^-olefin products and mediate the C_1_- to C_2_-transfer abilities of the enzyme, affording turnover rather than protein alkylation (28, 29). With this in mind, Tyr60 in 24-*Ac*SMT and corresponding Tyr64 in 28-*Ac*SMT were mutated to Phe or Leu and the resulting recombinant proteins expressed in *E. coli* in similar fashion to wild-type *Ac*SMT (supplemental Fig. S11). GC-MS analysis of the mutants, Tyr60Phe or Tyr60Leu, incubated with CTO, CHT, or ERGT showed products of molecular ion peaks of *m/z* 396 and 394, consistent with the M^+^ ions of ergosta-5,7,24(28)-trienol and ERGT, respectively, in yields ranging from 100% against CTO to 3–7% against CHT, while ERGT was recovered in unchanged form. Tyr64Phe and Tyr64Leu mutants incubated with CTO generated the same set of C_28_- and C_29_-steroidal products generated by wild-type, but in different proportions. In contrast to control 28-*Ac*SMT that converts CTO to C_28_- and C_29_-sterols in a ratio of 0:100 (supplemental Fig. S6), the Phe mutant produces a C_28_- to C_29_-sterol ratio of 16:84, while the Leu mutant produces a C_28_- to C_29_-sterol ratio of 82:18, showing the importance of Tyr64 in mediating the first and second C_1_-transfer reaction. A different picture emerges when CHT and ERGT are incubated with the Tyr64 mutants. As shown in Fig. 4F, G, Phe mutant produces an altered C_28_- to C_29_-sterol product ratio that includes blockage of C_28_-steroidal monol conversion and a significant reduction in inactivation products of the C_29_-steroidal diol pathway compensated by an increase in turnover products, including **18**, **19**, and **20**. Leu mutation led to the abolishment of activity such that ERGT was recovered in unchanged form, consistent with the importance of the aromatic residue in stabilizing cation-π interactions. The anomalous methylation increase in **20** by the Try64Phe mutant is presumably made possible by the relaxed control over substrate and intermediate conformations in the active site to favor the Δ^24(28)^-olefin pathway. The deprotonation leading to the formation of **20** may involve protons chemically and geometrically distinct from those lost in generation of the native substrate-24(28)-methylene lophenol, indicating that multiple amino acid residues (or peptide bonds) could be acting as adventitious active site bases in the mutant methyltransferase.

### CHT and ERGT are selective irreversible inhibitors of 28-*Ac*SMT

**Fig. 6.**
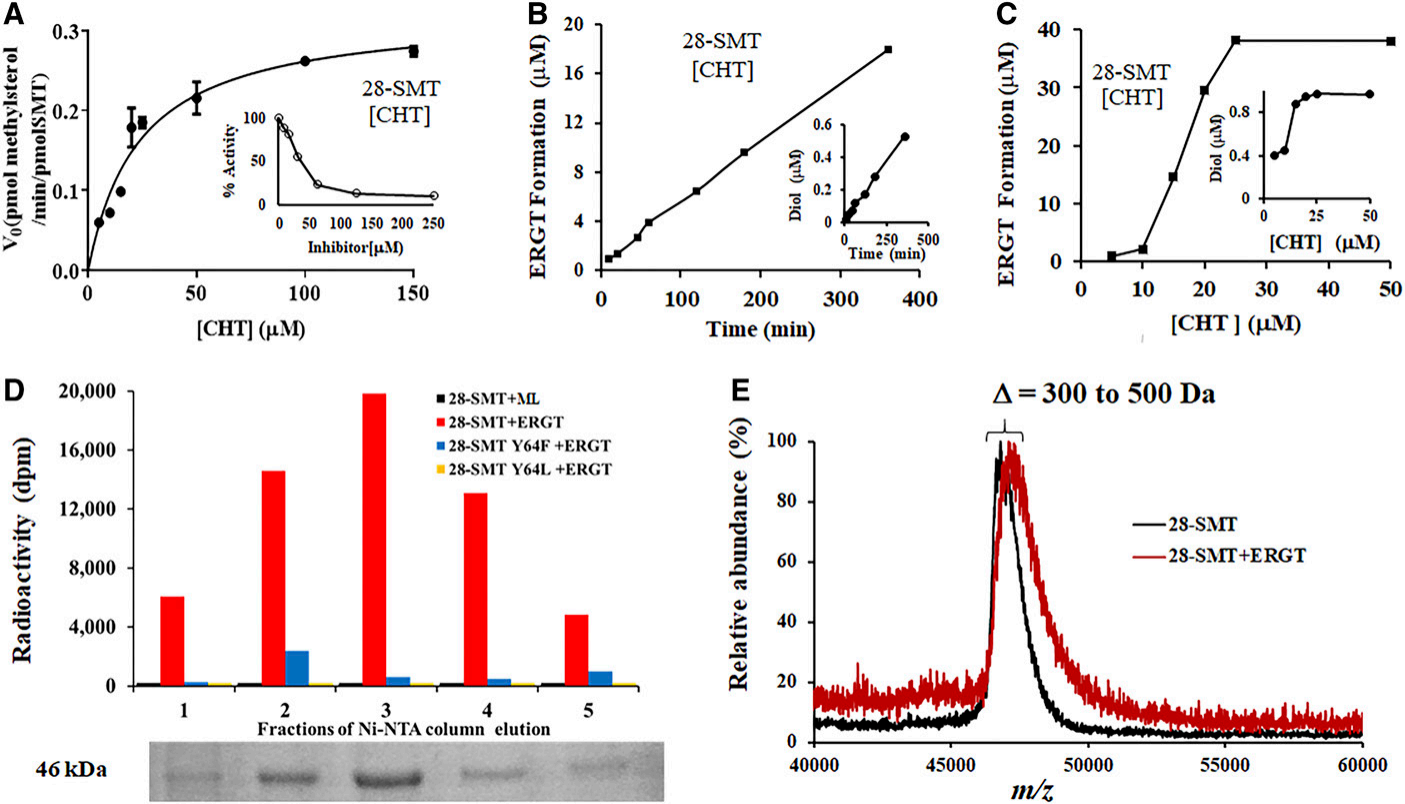
Kinetic, radiochemical, and proteomic analyses of yeast sterol analogs complexed with 28-*Ac*SMT. A: Plot of the initial velocity of a SMT-catalyzed reaction versus the substrate concentration (inset is the IC_50_ of CHT against the *K_m_* of 24(28)-methylene lophenol substrate). B: Plot of time of incubation against CHT conversion to C_28_-steroidal monol and diol. C: Plot of increasing concentration of CHT against product formation of C_28_-steroidal monol and diol. To limit C_29_-sterol formation, the SAM concentration is fixed at 100 μM (supplemental Fig. S12). D: SDS-PAGE gel of different Ni-NTA column fractions containing 28-*Ac*SMT complexed with ERGT versus radioactivity detected in each fraction (see the Materials and Methods for details of the binding experiment). F: Mass spectral analysis of ligand-free and ligand-bound (ERGT) 28-*Ac*SMT.

To investigate the requirement for covalence in a complementary fashion to that previously observed for 26,27-dehydrolanosterol against 24-*Ac*SMT where irreversible binding was established (supplemental Fig. S1C) (16), we studied the kinetics and mechanism-based covalent binding of 28-*Ac*SMT incubated with CHT and ERGT. In preliminary GC-MS analyses, we observed that CHT or ERGT incubated with 28-*Ac*SMT generated C_28_- and C_29_-steroidal diol products of molecular mass *m/z* 412 and 426, as expected for aborted methylation products of SMT turnover. Here, we evaluated CHT as substrate of 28-*Ac*SMT. CHT afforded a *K_m_* 25 μM and *k_cat_* 0.23 min^−1^ (**Fig. 6A**). These values compare with a *K_m_* 44 μM and *k_cat_* 1.5 min^−1^ for 28-*Ac*SMT assayed with its favored substrate, 24(28)-methylene lophenol. In a separate experiment, the concentration of CHT varied against fixed concentration (*K_m_*) 24(28)-methylene lophenol generated an IC_50_ 38 μM (*K_i_* 8 μM). For comparison purposes, the IC_50_ of CHT against cycloartenol incubated with 24-*Ac*SMT was determined to be 55 μM (*K_i_* 18 μM). The observed IC_50_s are comparable to those obtained for inhibition of protozoan and fungal SMTs by product analogs of the reaction.

We next incubated 28-*Ac*SMT with CHT paired with SAM at 100 μM in order to limit the second C_1_-transfer reaction (supplemental Fig. S12). SMT methylation of CHT yields multiple cations that when eliminated by deprotonation or quenched through protein alkylation led to a linear formation of C_28_-steroidal monol and diol (Fig. 6B, C). The 24(28)-methylene lophenol assayed at 50 and 100 μM concentrations of [^3^H_3_-*methyl*]SAM against CHT at 100 μM for 1 h was shown to protect the enzyme from inactivation, generating 10% and 25% C-methylation activity, respectively, relative to the C-methylation activity of a control incubation. These results indicate that inactivation is active site directed. Alternatively, no C_28_-steroidal diol is formed when 24-*Ac*SMT is incubated with CHT and no substrate protection observed when the experiment is carried out against cycloartenol as favored substrate.

Rate changes in sterol C24-methylation leading to distinct intermediate cations that convert to C_28_-steroidal monol and diol were used to establish the partition ratio. Thus, time course experiments of 28-*Ac*SMT assayed with initial concentrations of CHT at 0, 20, and 45 μM revealed the catalytic turnover of C24-methyl sterol monol formation (*r* = 0.99, Y = 0.0495x + 0.4001) to C24-methyl sterol diol formation (*r* = 0.88, Y = 0.0013x + 0.014) yield a partition ratio of 34 (Fig. 6B). This measure for formation of C24-methyl monol product per inactivation event is somewhat higher but no less deadly to the amoeba than the partition ratio of 3 established previously for 26-flourocholesta-5,7,24-trienol against the protozoan parasite, *Tb*SMT (15).

To verify covalent inhibition of 28-*Ac*SMT, we followed retention of the enzyme-generated ^3^H-intermediate bound to SMT through enzyme purification. Figure 6 shows that after Ni-NTA chromatography, neither wild-type SMT incubated with 24(28)-methylene lophenol nor mutant SMTs incubated with ERGT retain significant radioactivity in the homogenous protein, while wild-type SMT incubated with ERGT yields a complex of covalent binding (Fig. 6D). The irreversible nature of the ERGT-28-*Ac*SMT complex was further confirmed by MALDI-TOF analysis showing an increase in molecular mass of Δ300–500 Da (Fig. 6E), where the mass increase in the protein-sterol complex agrees with a single intermediate **22** forming the covalent enzyme-substrate adduct. These results support the GC-MS analysis of 28-*Ac*SMT mutants incubated with ERGT showing that either mutation shifted the partitioning away from the Δ^25(27)^-product necessary for proper C_28_- to C_29_-sterol balance and away from C_29_ steroidal diol formation indicative of protein alkylation.

### Steroidal antibiotic potency across kingdoms

**Fig. 7.**
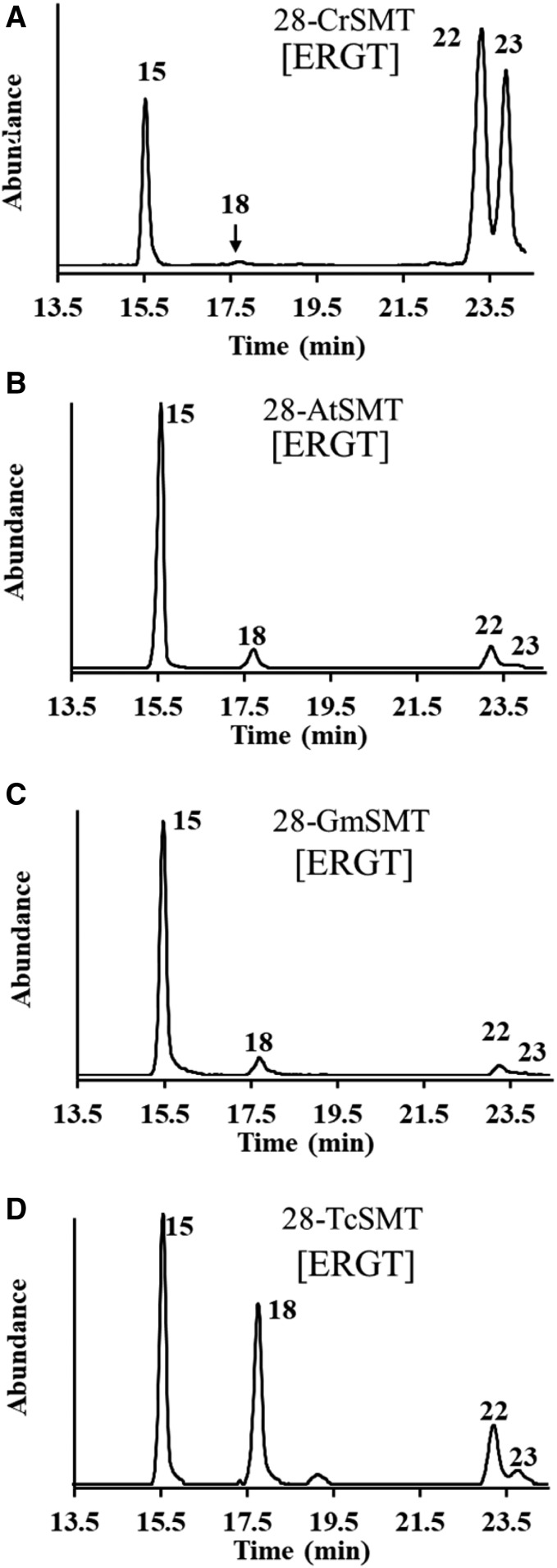
Sterol profiles of *Chlamaydomonas reinhardtii* (A), *Arabidopsis thaliana* (B), *Glycine max* (C), and *Trypanosoma cruzi* (D) SMT2-type enzymes incubated with ERGT. The compounds identified in the chromatograms are: **15**, ERGT; **18**, stigmast-5,7,22,24(28)-tetraenol; **22**, poriferasta-5,7,22(23)-trien-3,34-diol; and **23**, poriferasta-5,7,22(23)-trien-3,22-diol.

To determine whether the effect of the newly discovered steroidal antibiotics could be effective in regulating steroidogenesis beyond the amoeba, we examined ERGT as a substrate of multiproduct SMT2-type enzymes synthesized in green algae, land plants, and protozoa. The natural substrates of these enzymes are obtusifoliol (green alga SMT2), 24(28)-methylene lophenol (land plant SMT2), and ergosta-5,7,24(28)-trienol (trypanosome SMT2), which convert to 24-ethyl(idene)-Δ^24(28)^- or -Δ^25(27)^-olefin products (19, 30–33). Incubation of the four SMT2 enzymes with ERGT produced similar sterol profiles, as observed in the incubation of 28-*Ac*SMT with ERGT (**Fig. 7**). We observed SMT2 enzymes that favor the Δ^25(27)^-olefin pathway, as in the green alga and *Acanthamoeba* SMTs, produce significant C_29_-steroidal diol, suggesting extensive protein alkylation, while SMT2 enzymes that favor the Δ^24(28)^-olefin pathway, as in the trypanosome and land plant SMT2s, produce minor amounts of C_29_-steroidal diol, thereby minimizing the antimetabolite toxicity on C_29_-steroidogenesis. The different behavior of the SMT2 enzymes toward suicide substrate, as well as significant differences in sterol methylation reaction pathways, suggest species differences in the structures of the active site that employ distinct partitioning in response to an active-site-directed affinity label.

## DISCUSSION

SMT1 and SMT2 are the core components of phytosterol and mycosterol biosynthetic pathways and together are responsible for the generation of the vast array of 24-alkyl sterol structures. The fine-tuning of these methyltransferase enzymes under the pressure of natural selection can be correlated to sterol genealogy on the basis of substrate acceptance and the reactive carbocationic intermediate generated during methylation of the substrate Δ^24^-double bond (21, 31). As a result, initial substrate-enzyme complementarity to achieve a methylation-competent side chain conformation likely governs the specific reaction pathway to product variability and serves as driver of new 24-alkyl sterol traits. These findings together with the structural similarity in the primary sequences and tetrameric subunit organization of a wide-range of SMTs reflect common active-site topography and support the suggestion that these enzymes, each of strict substrate preference and catalyzing distinct canonical or branched reaction pathways in the construction of C_28_-ergostane and C_29_-stigmastane skeletons, respectively, evolved divergently from a common ancestral protein, presumed substrate promiscuous and catalytically diverse in product profile (6, 19).

With five examples of independently evolved SMT2 enzymes that show differential sensitivities to Δ^24^-monoene and Δ^22,24^-diene sterols represented by obtusifoliol, 24(28)-methylene lophenol, and ERGT (supplemental Fig. S2), it can be appreciated that lineage-specific steroidogenesis involving specific metabolites follows a common order of central catalysis where SMT2 enzymes seem to have shaped Δ^22^-C_29_-sterol production. When SMT2 enzyme cannot exercise sufficient control over productive side chain conformations for sterol methylation, catalysis is impaired and the normal course and equilibrium of metabolic sequences in steroidogenesis are inhibited, a consequence of which is to become an evolutionary dead end that prevents further expansion of C_29_-sterols. The process is seemingly hindered under physiological conditions, because the cell has adapted the biosynthetic sequence to avoid introduction of the Δ^22^-bond until after methylation of the Δ^24^-bond.

The substrate-specific SMT2 inactivation observed for CHT and ERGT of this work indicates a somewhat restricted binding region around the bound intermediate, and it is likely that the unique electronics and side chain orientation of Δ^22,24^-sterol analogs coordinated to sterol methylation of Δ^25(27)^-olefin production might have contributed to the SMT sensitivities that shaped sterol diversity early in evolution. Though land plant SMT1 enzymes can bind the substrate analog, 24-methyl cycloartanol (= cyclobranol) (34, 35), the analog tested against soybean SMT1 led to enzyme inactivation (35). Typically in land plants, cycloartenol is converted to 24-methyl desmosterol, which is converted by the sterol 24-reductase to campesterol (24α-methyl cholesterol). Intriguingly, 24-methyl desmosterol binds nonproductively to SMT1 showing the importance of substrate acceptability against steroidal enzymes competing for the side chain Δ^24^-bond (2, 34). Thus, as for CHT and ERGT capable to destroy *Acanthamoeba* SMT2 cyclobranol presents side chain features to land plant SMT1 that can terminate the enzyme in a time-dependent manner (35). It is worth mentioning that the sterol methylation reaction for the amoeba and land plant SMT differ; one operates the ∆^25(27)^ -olefin pathway while the other operates the Δ^24(28)^ -olefin pathway, suggesting mechanistic specificity toward protein alkylation is linked to the enzyme recognition of substrate shape and structure of the functional group. Consequently, when these particular side chains are coupled to an ineffective substrate, then metabolites are produced out of order, thereby compromising steroidogenesis. While still fragmented, these new observations of SMT acceptance of potential steroidal antimetabolites depict individual patterns of metabolite order in sterol biosynthesis (Fig. 1) may have evolved in response to specific enzyme sensitivities for suicide substrates. We surmise that, if the SMT reactions operating far from equilibrium dictate the direction and regulatory capacity of the sterol metabolic pathway, then successful catalysis is not random but requires specific partnering of substrate with SMT enzyme.

By following a lead from synthetic suicide substrates that can inactivate SMT and knowledge of function of aromatic amino acids in the substrate binding segment, a specific tyrosine64 to phenyalanine change in 28-*Ac*SMT (SMT2) was found to be sufficient to essentially switch the reaction channeling from one that inhibits catalysis in the presence of ERGT to one that enables protein to synthesize utilizable products used in remodeling the C_28_- to C_29_-sterol profile during the course of evolution. The promiscuity of such single mutants at position-64 originating from the altered methylation template could have potentiated evolution of the ancestral protein, leading to the production of Δ^22,24^-substrates. Many of the SMT1 enzymes, such as in fungi, soybean, Arabidopsis, and amoeba, are high-fidelity SMTs that generate one product rapidly through a nonstop methylation-deprotonation reaction coupled to the Δ^24(28)^-olefin pathway. However, SMT2 enzymes, such as in green algae, land plants, protozoa, and amoeba, generate one major methyl product and (usually) minor quantities of one or more isomeric methyl products through a step-wise mechanism that proceeds through the ∆^25(27)^ - or ∆^24(28)^ - olefin pathways. Thus, when SMT2s accommodate a more permissive substrate, the enzyme-generated intermediate can undergo premature quenching and, according to off-pathway conformations, fail to convert productively. For these reasons, Δ^22,24^-substrates shown to be catalytically productive against different SMT2-type enzymes cannot prevail in C_29_-sterol biosynthesis pathways. It is worth mentioning that the work described here provides long-sought direct evidence for the first 24-ethyl intermediate in SMT2 catalysis represented by compound **22** that converts to multiple 24-ethyl(idene) products (36, 37).

A surprising discovery was the observation of turnover-dependent inactivation of 28-*Ac*SMT in vivo, suggesting that the sterol methylation catalyst generating C_29_-sterols could be a select target in antibiotherapies, while the natural products could be a new class of antibiotic considered to be biosynthetic antimetabolites. These newly identified antimetabolites that can masquerade as essential metabolites and promote catalytic destruction may now be considered natural poisons of select parasitic organisms. The sterol analysis of treated Ac cells cultured in T-flasks indicated that the antimetabolites inhibit overall steroidogenesis. However, the slight increase in C_29_-sterol in 96 h-treated cells suggests a metabolic response of overexpression of SMT2 enzyme, consistent with the long exposure of the suicide substrate on target enzyme.

Although CHT can outcompete the natural substrate and inhibit 24-*Ac*SMT and 28-*Ac*SMT with similar *K_i_* values, the loss of inactivation power against 24-*Ac*SMT can be attributed to the catalytic Δ^24(28)^-olefin pathway operated by most SMT1-type enzymes (33, 37). The in vitro potency of the CHT established via the ratio of the kinetic values *K_m_* of natural substrate/*K_i_* of inhibitor against 28-*Ac*SMT of 25 μM/8 μM = 3, compare favorably with the in vivo amoebicidal potency of 5 μM. Interestingly, the thrust of CHT-induced inactivation appears to come from inhibition of the C_29_ reaction intermediate trapped by an active site base. Mechanistically, extended conjugation of Δ^22^-bond in Δ^24^-substrates exerts a dramatic increase in the reactivity of substrate by increasing the transition state for formation of the 24-methyl/ethyl C24-cation intermediate. This structure becomes a transition-state/reactive intermediate analog affording the suicide substrate represented by E-I* species that proceeds down path a in **Scheme 1**. In contrast, the transition state/high energy intermediate analogs represented by 25-azacycloartanol undergo reversible (albeit tight) binding as indicated in Scheme 1, where E-I ultimately returns to E + I.

Scheme 1. Kinetic scheme for inhibition of *Ac*SMT by a substrate analog. 1: Binding of enzyme (E) and Δ^24^-sterol substrate (S) followed by enzyme catalysis with formation of methyl product (P). 2: Inhibitor (I) binds to enzyme followed by activation via methylation (E-I*) and this intermediate can partition along path b and dissociate from the active site (E+P) or enter into path a that leads to irreversible binding (E-I*) (adapted from Ref. 16).
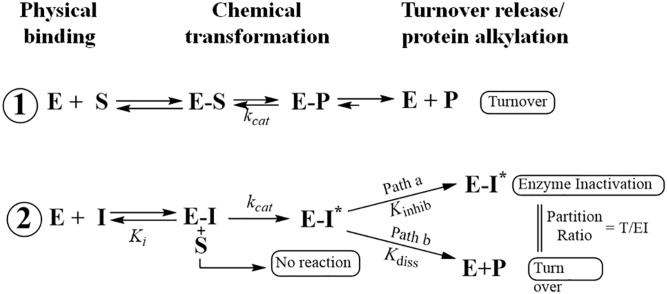


In summary, we have succeeded in identifying a new class of antibiotic, elucidating its mode of action and uncovering the origin of its remarkable specificity. This work highlights the importance of using a combination of chemical, enzymatic, and microbiological approaches (38) in antimetabolite discovery and reveals a conceptually elegant mechanism of action. Encouraged by the steroidal antimetabolite depriving the parasite of essential sterol products without effect on the host and then to show the antimetabolite acts as a catalytically incompetent dead-end analog, we anticipate that CHT and ERGT will serve as prototype antibiotic inhibitors that will enable further preclinical validation and will inspire a search for a wider range of steroidal antimetabolites targeting alternate sterolic enzymes that could be developed into leading pharmaceuticals. In order to gain insight into the translational aspects of the current research, the portfolio of protozoan diseases needs to be further expanded, and comparison of in vitro assays, animal models of infection, and clinical data must be performed with the new steroidal antibiotics, all of which are currently underway.

## Supplementary Material

Supplemental Data
