# Supplementary material for: Steroidal antibiotics are antimetabolites of Acanthamoeba steroidogenesis with phylogenetic implications
Source: J Lipid Res. 2019 Feb 1;60(5):981–94. doi: 10.1194/jlr.M091587 (PMC6495176; doi:10.1194/jlr.M091587)
Supplement: Supplemental Data [file supp_60_5_981__index.html]

Steroidal antibiotics are antimetabolites of Acanthamoeba steroidogenesis with phylogenetic implications — Steroidal antibiotics are antimetabolites of Acanthamoeba steroidogenesis with phylogenetic implications — Supplemental Data 

# Steroidal antibiotics are antimetabolites of Acanthamoeba steroidogenesis with phylogenetic implications

## Supplemental Data

- Supplemental Figures (.docx, 4.7 MB) - Supp Files
