# Supplementary material for: Steroidal antibiotics are antimetabolites of Acanthamoeba steroidogenesis with phylogenetic implications
Source: J Lipid Res. 2019 Feb 1;60(5):981–94. doi: 10.1194/jlr.M091587 (PMC6495176; doi:10.1194/jlr.M091587)
Supplement: Supplemental Data [file 10.1194_M091587_jlr.M091587-1.docx]

**Supplemental Figures (12) and Tables (2)**

Steroidal antibiotics are antimetabolites of Acanthamoeba steroidogenesis with phylogenetic implications.

Wenxu Zhou, Emilio Ramos, Xunlu Zhu, Paxtyn M. Fisher, Medhanie E. Kildane, Boden H. Vanderloop, Crista D. Thomas, Juqiang Yan, Ujjal Singha, Minu Chaudhuri, Michael T. Nagel, and W. David Nes


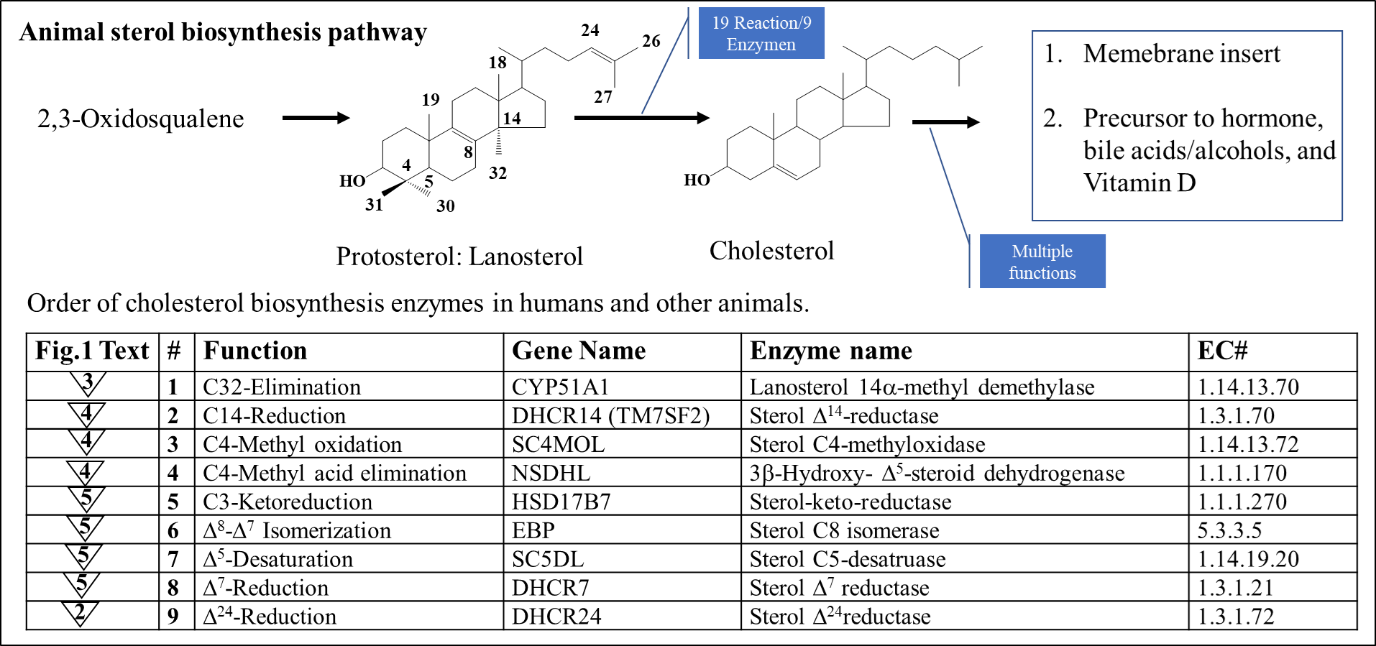


**Supplemental Figure 1A**. Cholesterol biosynthesis pathway in animals. Note- the multiple functions for cholesterol are not exhaustive and others could include covalent lipidation of proteins. The order of sterol enzymes presented here follow the “Bloch” pathway.


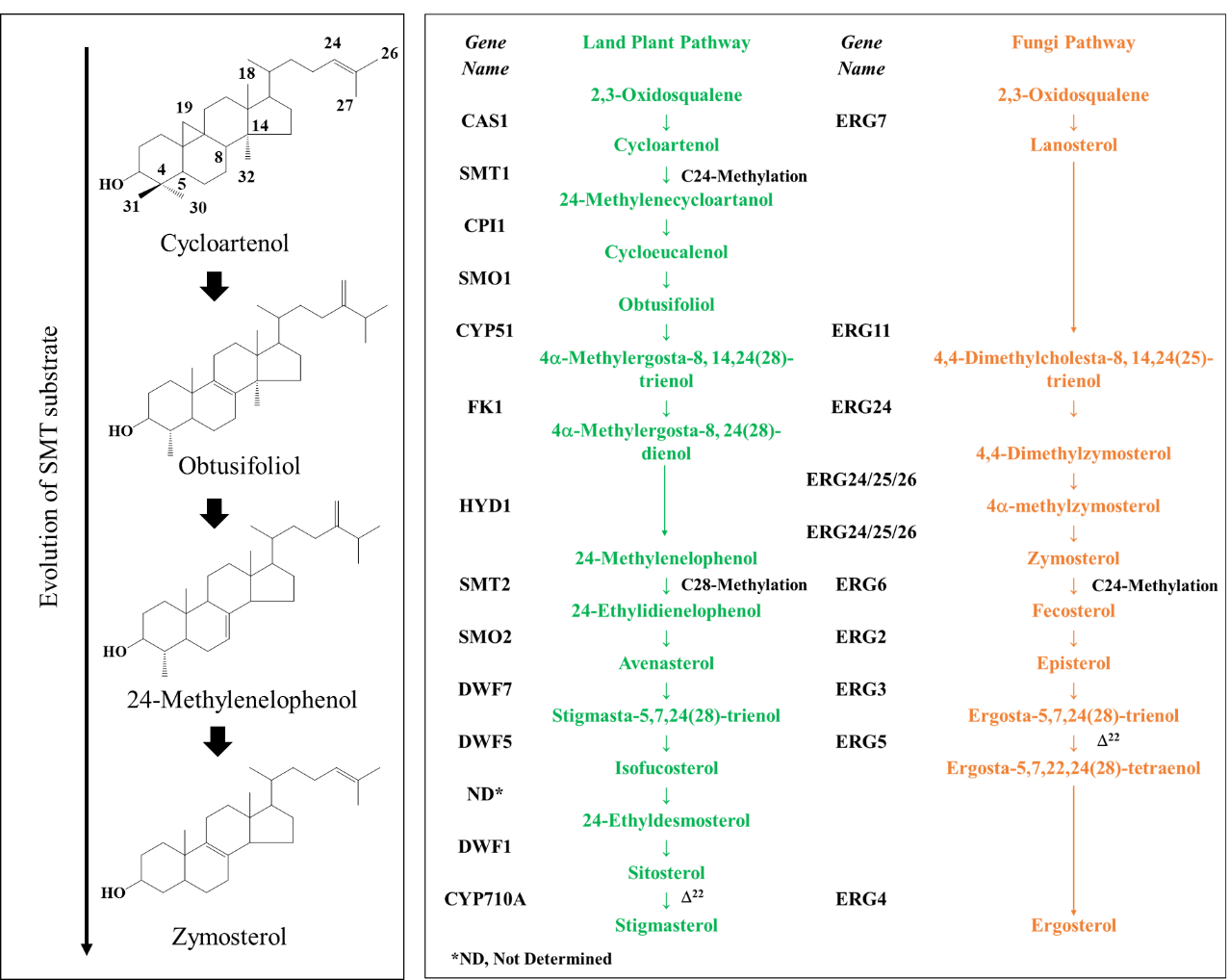


**Supplemental Figure 1B**. Phylogenetic characterization of sterol biosynthesis enzymes outlined as they are reported to appear in land plants and fungi. Adapted from reference 9 and 10. Note late positioning of Δ^22^-bond introduction into sterol biosynthesis pathway and the natural occurrence of ERGT as a final intermediate in the ergosterol biosynthesis pathway.

**Supplemental Figure 1C**. Sterol methylation reaction pathways subject to substrate recognition that lead to turnover or protein alkylation. N = sterol nucleus.

**Supplemental Figure 2**. Structures of sterols studied herein- also see Supplemental Table 1.


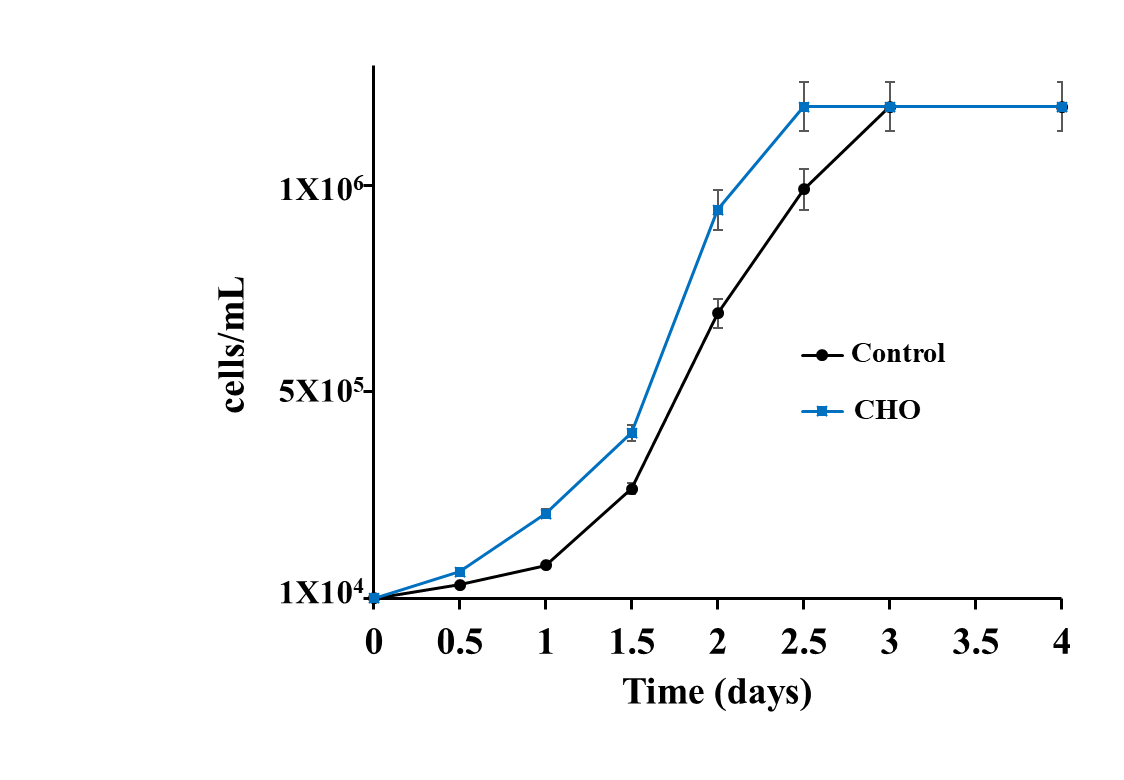


**Supplemental Figure 3**. Growth curve of *A. castellanii* trophozoites cultured on fetal bovine serum containing 10 µM cholesterol or control in 25 ml T-flasks supplemented with 5 ml medium.


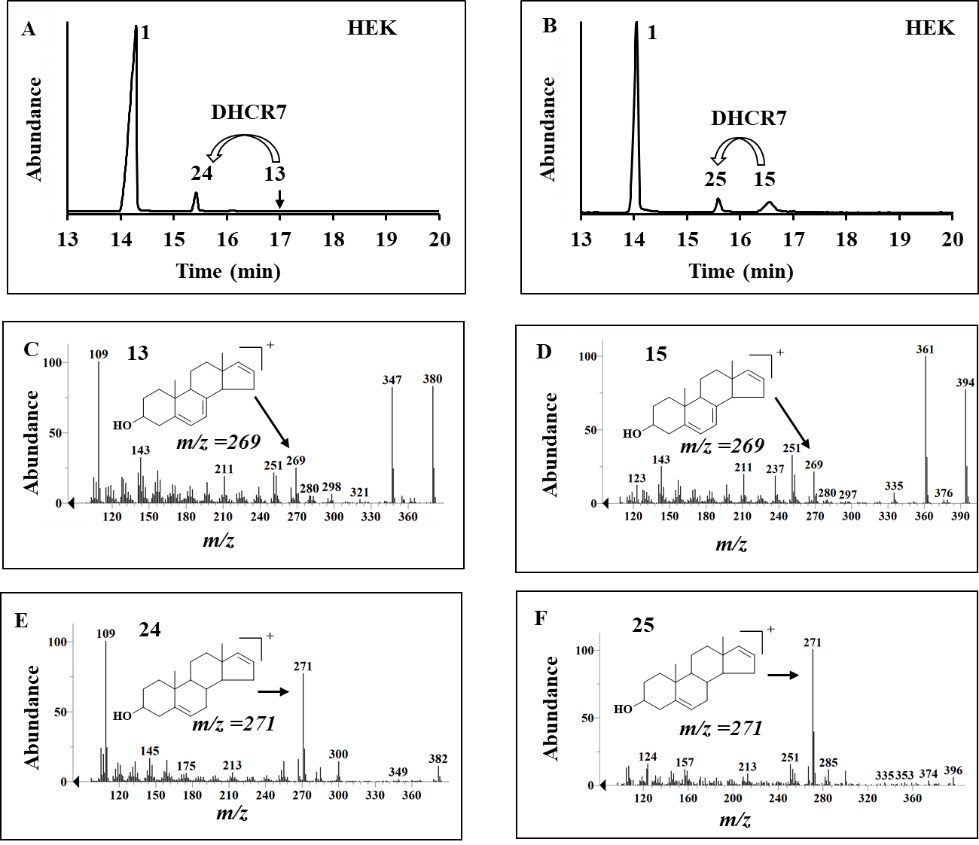


**Supplemental Figure 4.** Chromatographic and mass spectral analysis of HEK cell total sterol from incubation of CHT (Panels A, C and E) or ERGT (Panels B, D and F).


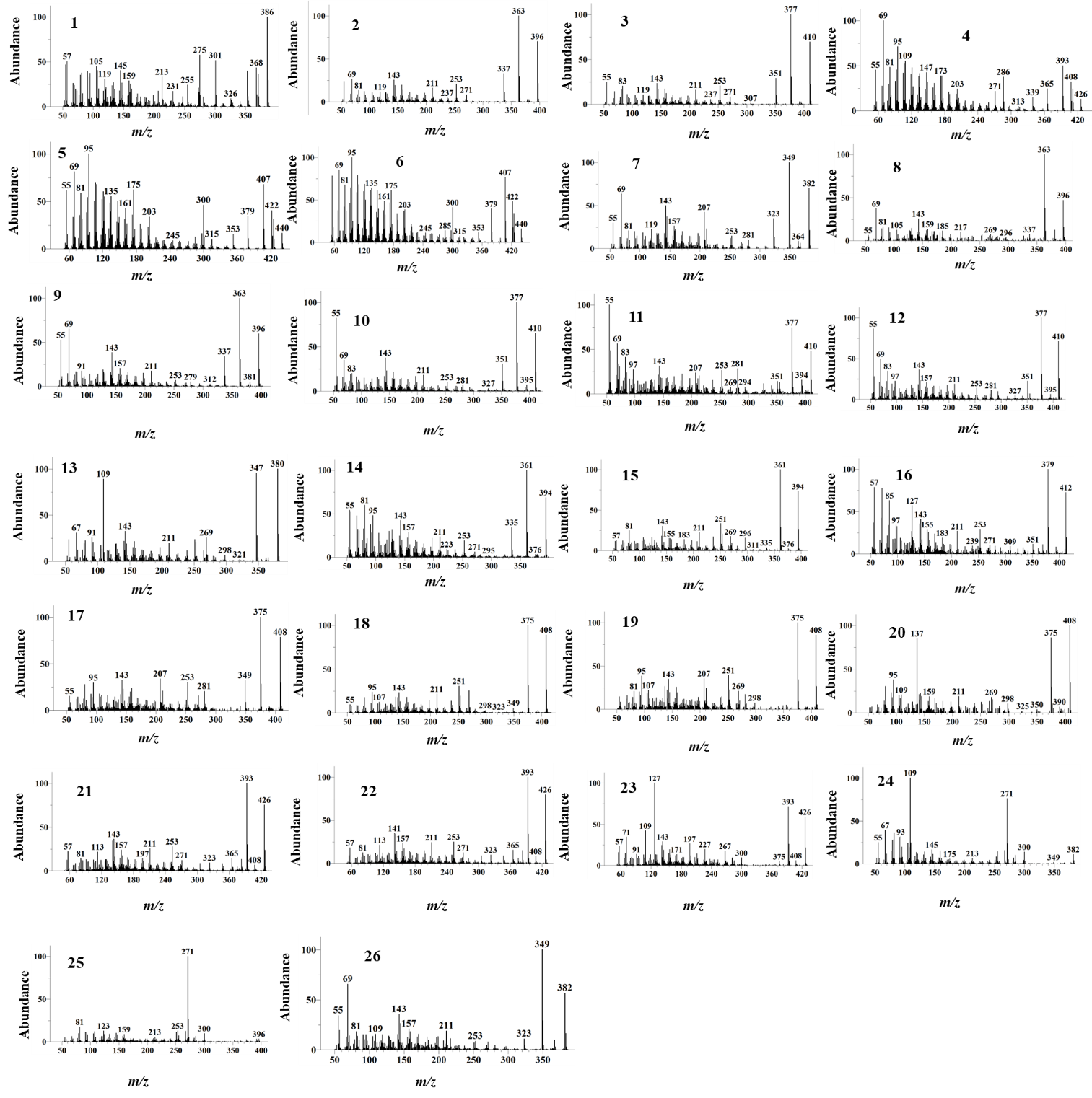


**Supplemental Figure 5**. Mass spectra of sterols identified in the current studies- Also see Supplemental Table 1.


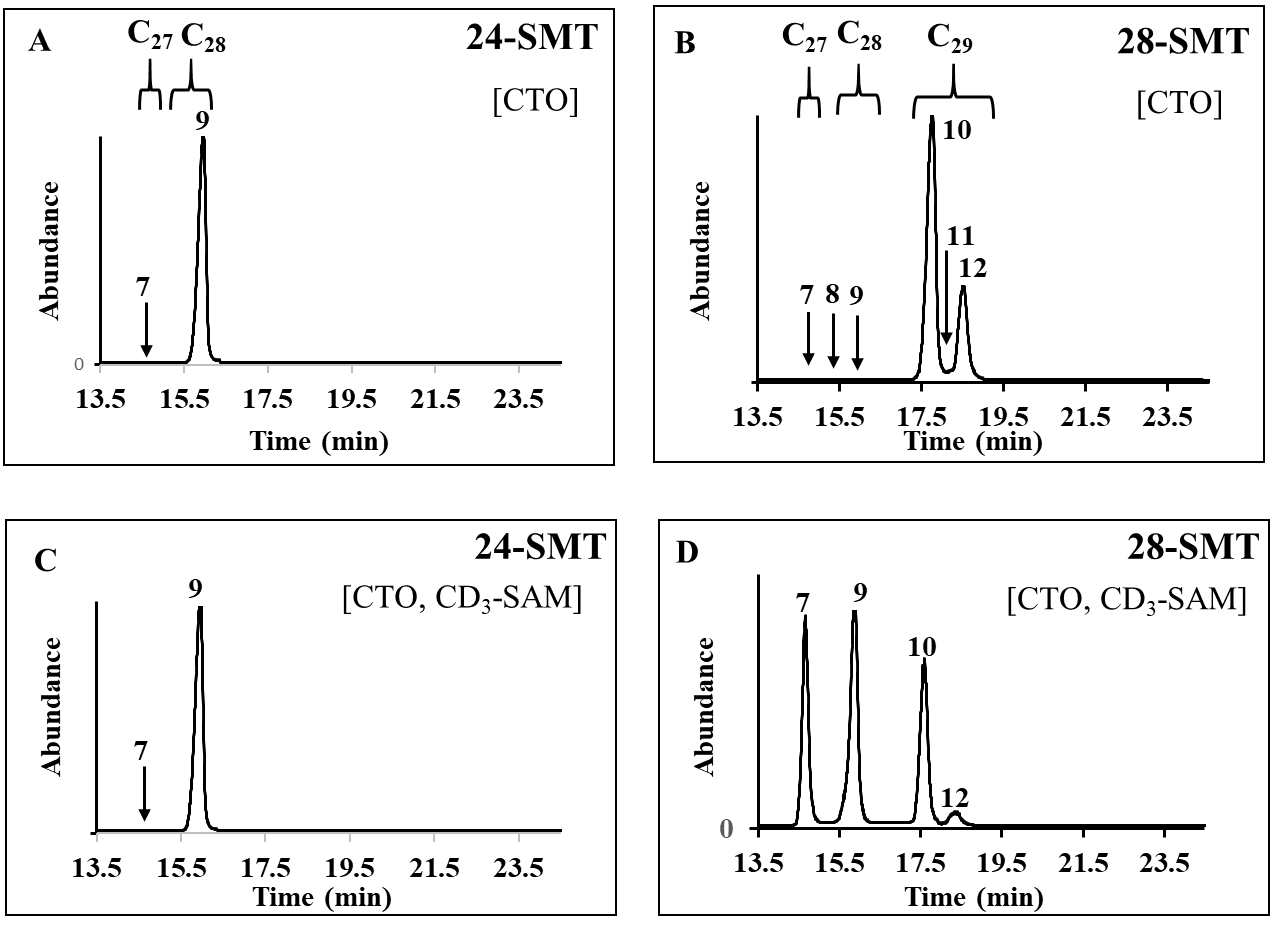


**Supplemental Figure 6.** GC profile of 24-AcSMT or 28-AcSMT incubated with cholesta-5,7,24-trienol paired with SAM or [^2^H_3_-*methyl*]SAM = CD_3_-SAM.


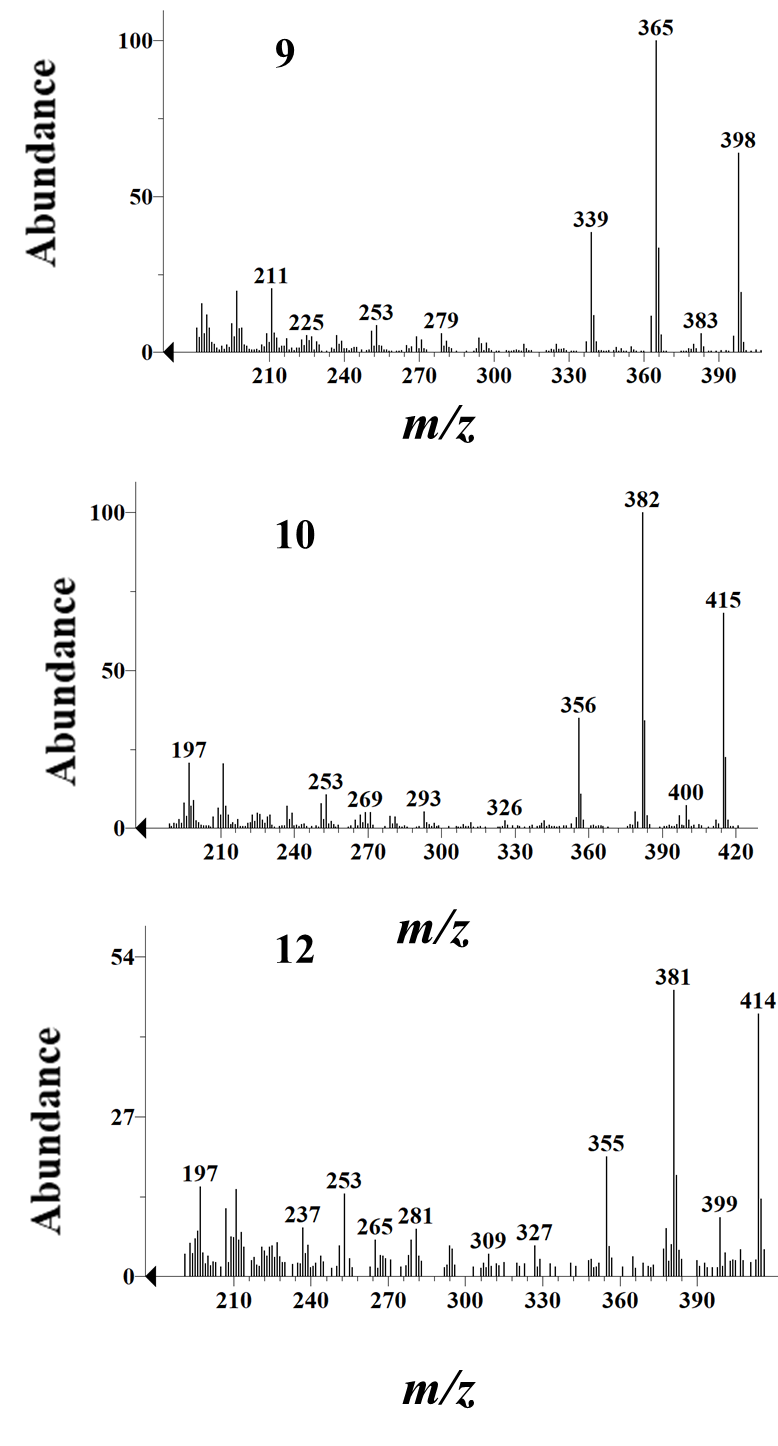


**Supplemental Figure 7**. Mass spectra of C_28_- and C_29_-sterols labeled with deuterium at C28 and C29 from incubation of 28-*Ac*SMT with CTO and [^2^H_3_-*methyl*]SAM (refer to GC in Supplemental Figure 6, Panel D).


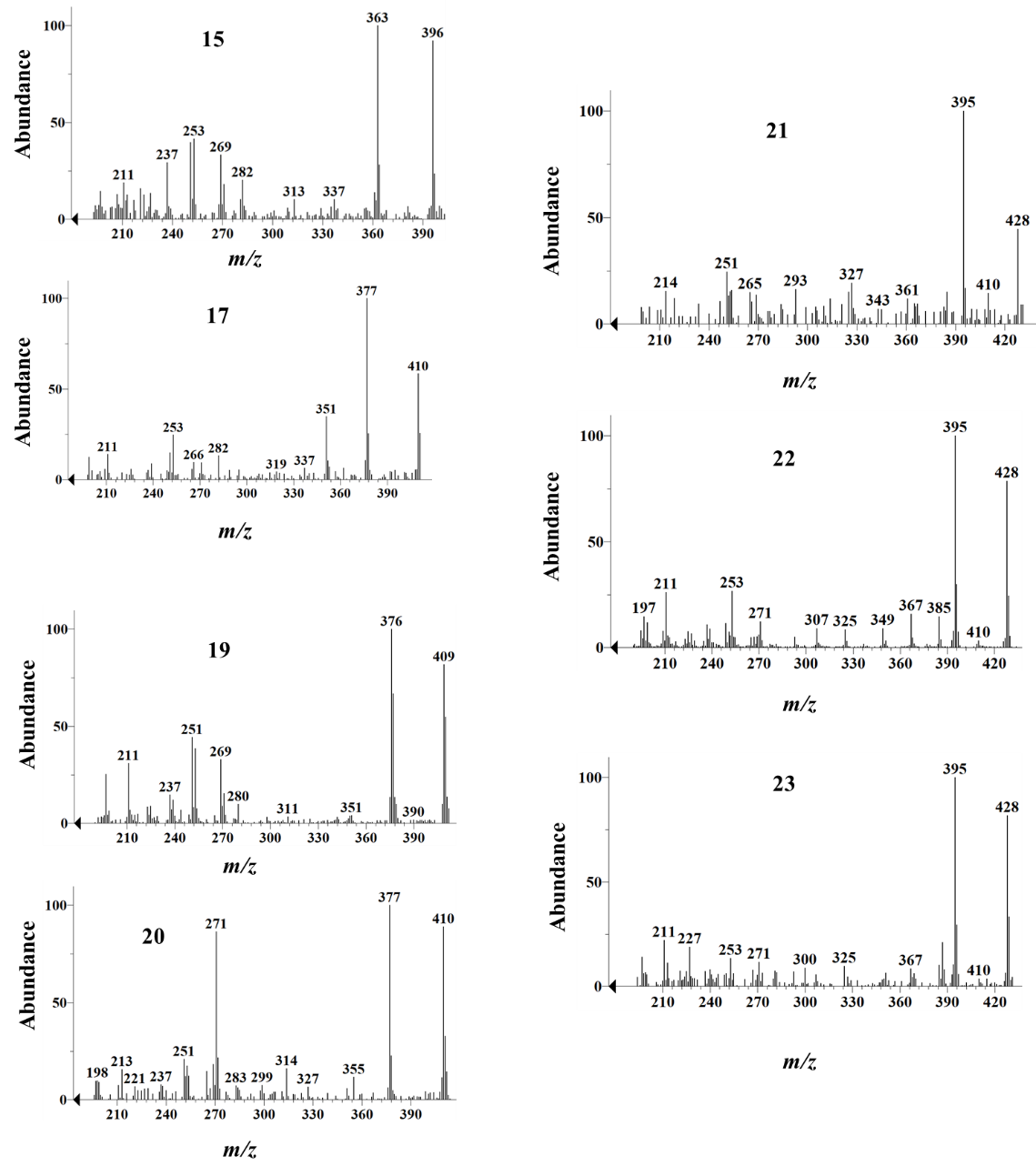


**Supplemental Figure 8**. Mass spectra of C_28_- and C_29_-sterols labeled with deuterium at C28 from incubation of 28-AcSMT with [28-^2^H]ERGT and SAM.


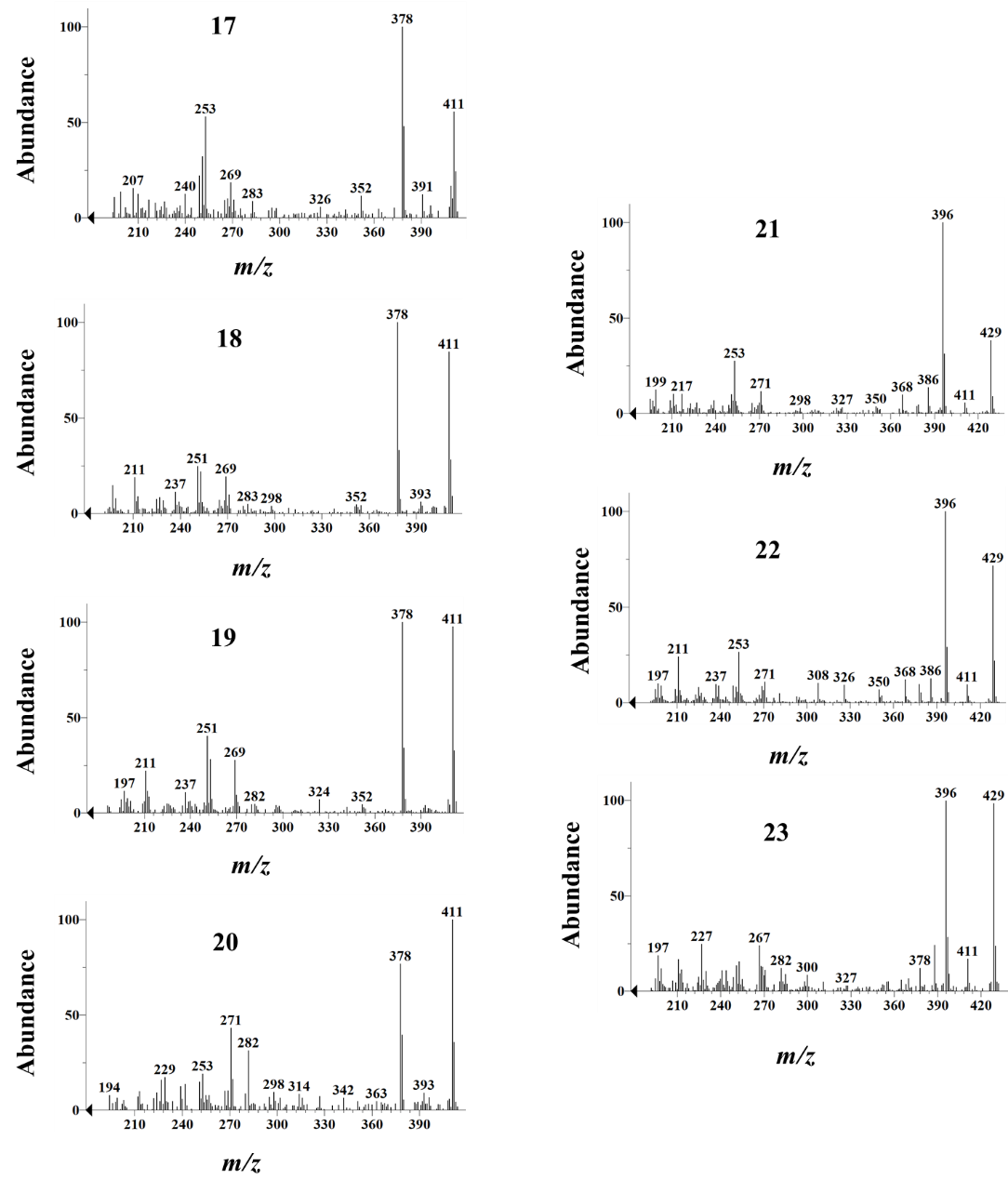


**Supplemental Figure 9**. Mass spectra of C28 and C29- sterols labeled with deuterium at C29 from incubation of 28-*Ac*SMT with ERGT and [^2^H_3_-*methyl*]SAM.


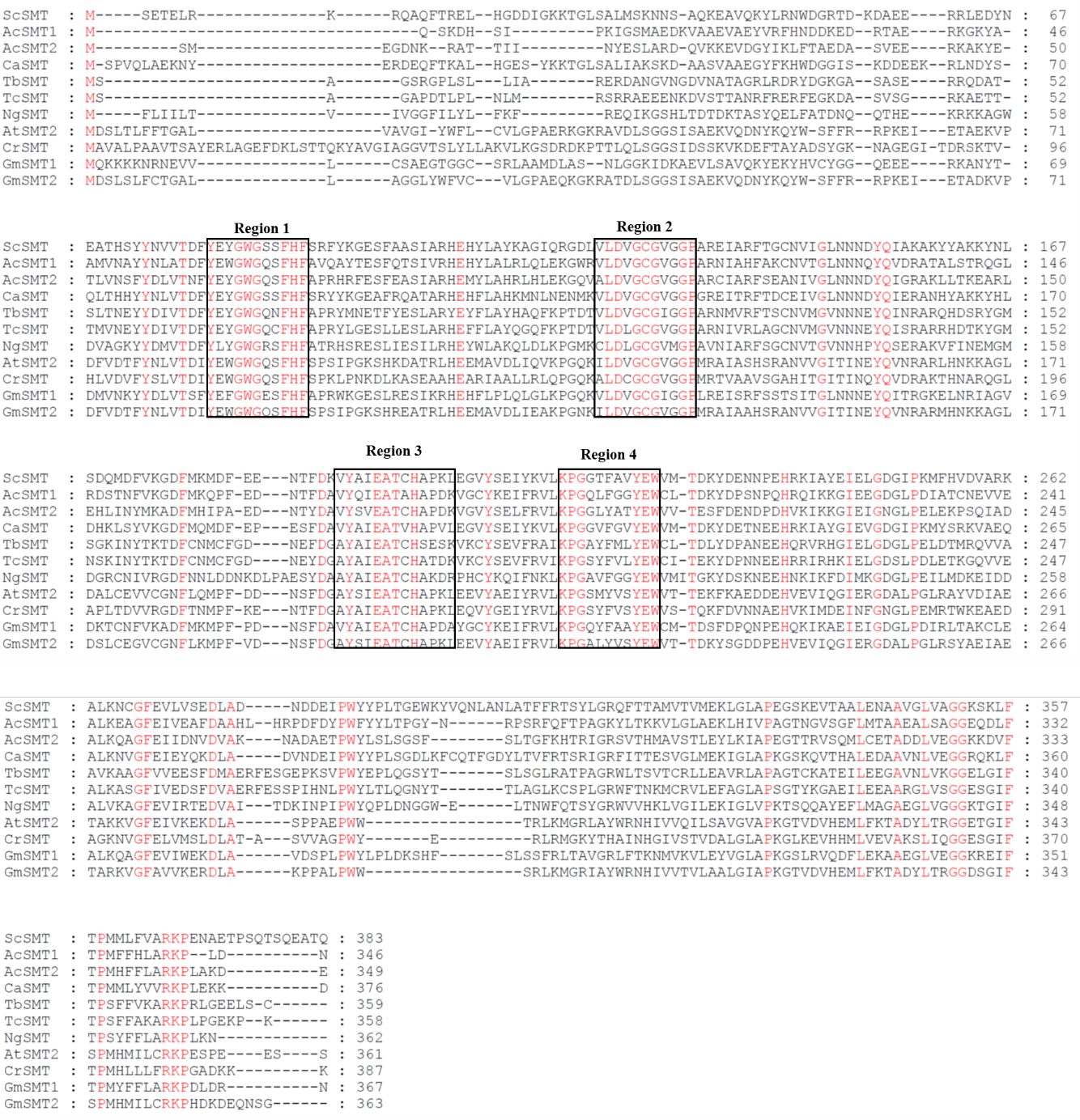


**Supplemental Figure 10**. Amino acid sequence overlay for sterol methyltransferase enzymes previously cloned and purified in the Nes laboratory (16, 25,27,28,29 and additional references cited therein). Relevant regions for substrate binding segments of sterol (I, III, IV) and SAM (II) in Regions 1 to IV are illustrated.

SMTs cloned are: Sc-Sacharomyces cersevisiae, Ac, Acanthamoeba castellanii, Ca, Candida albicans, Tb, Trypanosoma brucei, Tc, Trypanosoma cruzi, Ng, Naegleria gruberi, At, Arabidopsis thaliana, Cr, Chlamydomonas reinhardtii, and Gm, Glycine max.


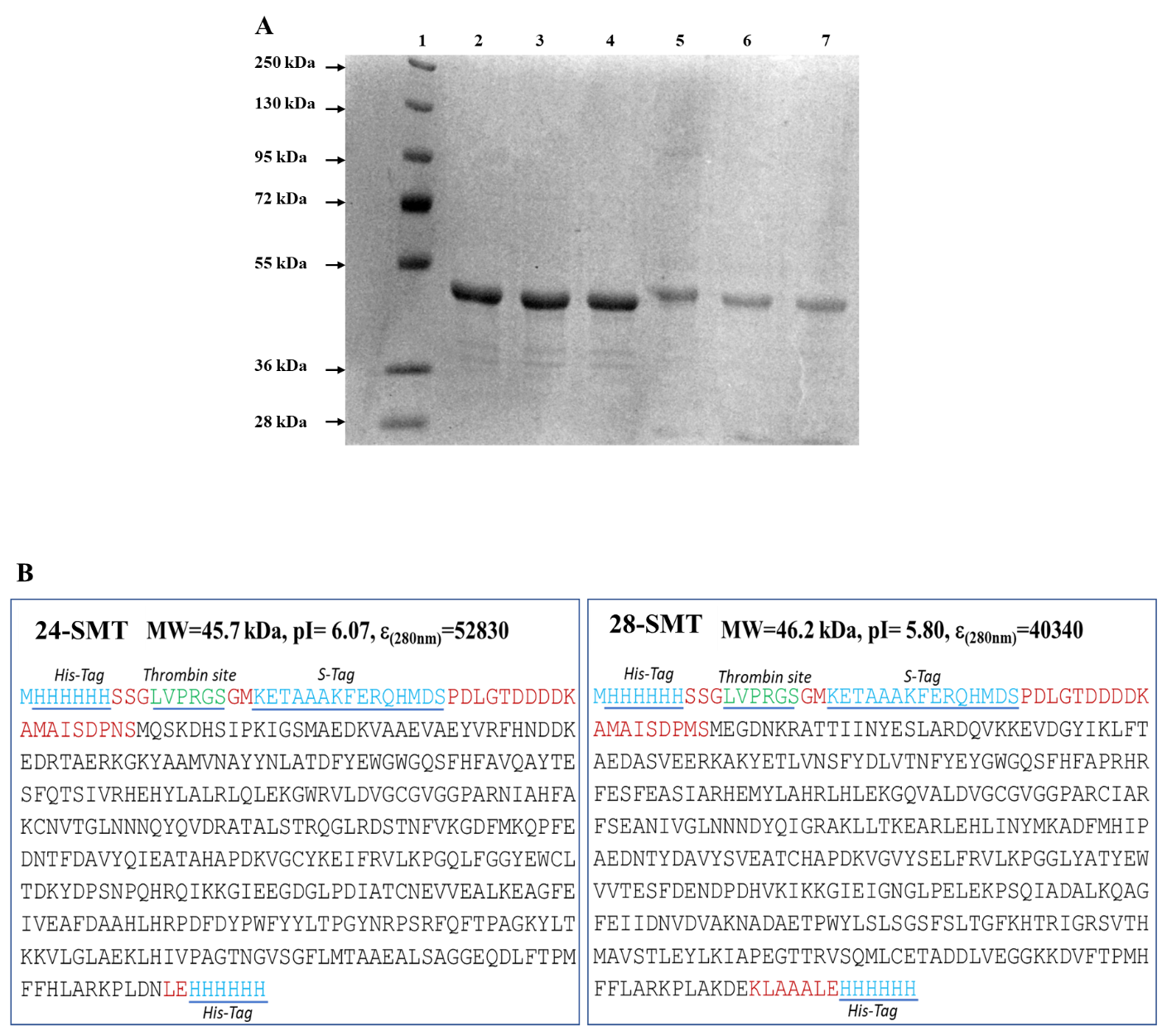


**Supplemental Figure 11.** SDS-PAGE gel analysis of pure His_12_-tagged wild-type and mutant *Ac*SMTs. Panel A: Lane 1- standards, Lanes 2 and 5 are wild-type 24-AcSMT and 28-AcSMT, respectively. Lanes 3,4 6, and 7 are of Tyr to Phe or Leu replacement in 24-AcSMT or 28-AcSMT, respectively. Panel B: Proteomic analysis of pure His_12_-tagged 24-AcSMT and 28-AcSMT that confirms identities of cloned protein.


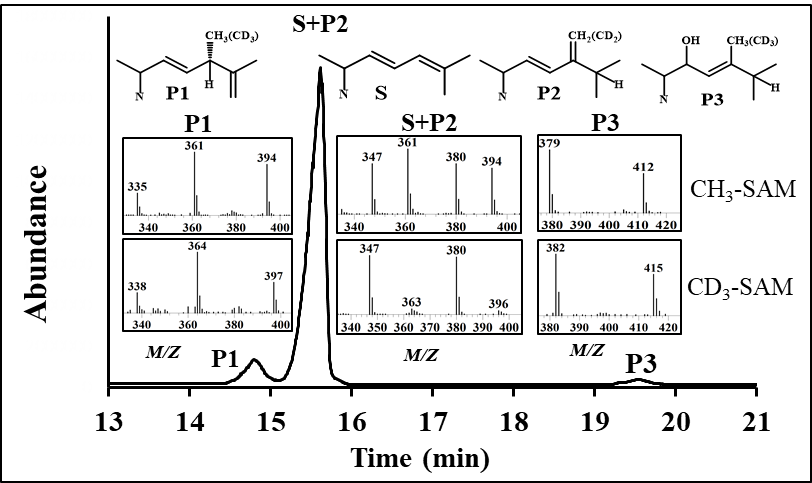


**Supplemental Figure 12**. GC-MS analysis of total sterol products from overnight 28-*Ac*SMT incubation of CHT (100 µM) and SAM or [^2^H_3_-*methyl*]SAM (100 µM). S= substrate of CHT, P = enzyme-generated product as indicated in the figure. P1=**14**, P2= **15**, P3= **16** and S = **13**. See Supplemental Figure 2 for a key to structures.

STable 1. Chromatographic and spectral constants of sterols studied against Acanthamoeba^1^.

| Sterol | Carbon number | Structure | MW (M^+^) | GC *(RRTc*) | UV(λ_max_, nm) | TLC (R_f_) |
| --- | --- | --- | --- | --- | --- | --- |
| Cholesterol | C_27_ | 1 | 386 | 1.00 | 210 | 0.33 |
| Ergosterol (ERG) | C_28_ | 2 | 396 | 1.13 | 282 | 0.33 |
| 7-dehydroporiferasterol (DHP) | C_29_ | 3 | 410 | 1.32 | 282 | 0.33 |
| Cycloartenol (CA) | C_30_ | 4 | 426 | 1.43 | 210 | 0.43 |
| 24(28)-methylene cycloartanol | C_31_ | 5 | 440 | 1.59 | 210 | 0.38 |
| Cyclolaudenol | C_31_ | 6 | 440 | 1.57 | 210 | 0.43 |
| Cholesta-5,7,24-trienol (CTO) | C_27_ | 7 | 382 | 1.13 | 282 | 0.33 |
| Ergosta-5,7,25(27)-trienol | C_28_ | 8 | 396 | 1.12 | 282 | 0.33 |
| Ergosta-5,7,24(28)-trienol | C_28_ | 9 | 396 | 1.14 | 282 | 0.33 |
| Poriferasta-5,7,25(27)-trienol | C_29_ | 10 | 410 | 1.28 | 282 | 0.33 |
| Stigmasta-5,7,24(28)*E*-trienol | C_29_ | 11 | 410 | 1.30 | 282 | 0.33 |
| Stigmasta-5,7,24(28)Z-trienol | C_29_ | 12 | 410 | 1.34 | 282 | 0.33 |
| Cholesta-5,7,22,24-tetraenol (CHT) | C_27_ | 13 | 380 | 1.13 | 282 | 0.33 |
| Ergosta-5,7,22,25(27)-tetraenol | C_28_ | 14 | 394 | 1.07 | 282 | 0.33 |
| Ergosta-5,7,22,24(28)-tetraenol (ERGT) | C_28_ | 15 | 394 | 1.13 | 232/282 | 0.33 |
| Ergosta-5,7,23-trien-3,22-diol | C_28_ | 16 | 412 | 1.42 | 282 | 0.23 |
| Protothecasterol | C_29_ | 17 | 408 | 1.21 | 282 | 0.33 |
| Stigmasta-5,7,22, 24(28)*E*-tetraenol | C_29_ | 18 | 408 | 1.27 | 232/282 | 0.33 |
| Stigmasta-5,7,22, 24(28)*Z*-tetraenol | C_29_ | 19 | 408 | 1.28 | 232/282 | 0.33 |
| Poriferasta-5,7,20(22),23-tetraenol | C_29_ | 20 | 408 | 1.38 | 230/282 | 0.33 |
| Poriferasta-5,7,22-trien-3,25-diol | C_29_ | 21 | 426 | 1.62 | 282 | 0.23 |
| Poriferasta-5,7,22-trien-3,24-diol | C_29_ | 22 | 426 | 1.68 | 282 | 0.23 |
| Poriferasta-5,7,22(23)-trien-3,22-diol | C_29_ | 23 | 426 | 1.73 | 282 | 0.23 |
| Cholesta-5,22,24-trienol | C_27_ | 24 | 382 | 1.08 | 210/230 | 0.33 |
| Ergosta-5,22,24-trienol | C_28_ | 25 | 396 | 1.11 | 210/230 | 0.33 |
| Cholesta-5,7,22-trienol | C_27_ | 26 | 382 | 1.04 | 282 | 0.33 |

^1^ Analytics described in the Method section.

| MUTATION | PRIMER | CODON CHANGE |
| --- | --- | --- |
| 24-SMTY60Ff | ctggccaccgacttttttgagtggggctg | TAT to TTT |
| 24-SMTY60Fr | cagccccactcaaaaaagtcggtggccag |  |
| 24-SMTY60Lf | caacctggccaccgactttttagagtggggctg | TAT to TTA |
| 24-SMTY60Lr | cagccccactctaaaaagtcggtggccaggttg |  |
| 28-SMTY64Ff | cgatctcgtgaccaacttcttcgagtacggct | TAC to TTC |
| 28-SMTY64Fr | agccgtactcgaagaagttggtcacgagatcg |  |
| 28-SMTY64Lf | ctcgtgaccaacttcttagagtacggctggggtc | TAC to TTA |
| 28-SMTY64Lr | gaccccagccgtactctaagaagttggtcacgag |  |

S Table 2. Mutagenic primers for AcSMT.
